# Optical and THz investigations of mid-IR materials exposed to alpha particle irradiation

**DOI:** 10.1038/srep40209

**Published:** 2017-01-09

**Authors:** Dan Sporea, Laura Mihai, Adelina Sporea, Ion Vâţã

**Affiliations:** 1 National Institute for Laser, Plasma and Radiation Physics, Center for Advanced Laser Technologies, Magurele, RO-077125, Romania; 2 “Horia Hulubei” National Institute of Physics and Nuclear Engineering, RO-077125, Magurele, Romania

## Abstract

The paper is the first comprehensive study on alpha particle irradiation effects on four mid-IR materials: CaF_2_, BaF_2_, Al_2_O_3_ (sapphire) and ZnSe. The measurements of the optical spectral transmittance, spectral diffuse reflectance, radioluminescent emission, terahertz (THz) spectral response, transmittance, absorbance, refractive index, real and imaginary parts of the dielectric constant and THz imaging are used as complementary investigations to evaluate these effects. The simulations were run to estimate: (i) the penetration depth, (ii) the scattering of alpha particle beam, (iii) the amount of material affected by this interaction, and (iv) the number of vacancies produced by the radiation exposure for each type of material. The simulation results are compared to the off-line measurement outcomes. The delay and spectral composition change of the reflected THz signal highlight the modification induced in the tested materials by the irradiation process.

Devices and equipments included as space missions constituents are subjected to energetic radiation fields such as^1^,^2^,^3^,^4^: solar flare, trapped protons, trapped electrons, galactic cosmic ray – GCR (protons, alpha particles, heavy ions), radiation which might affect their normal operation. Radiation effects in materials and components are extensively considered in the last 10 years as the incidents associated with their destruction/degradation are more and more complex and expensive to circumvent during a mission, affecting normal operation, inducing components and instruments malfunction or producing major interferences in sensing systems. Designing special systems for space borne applications became an issue because of higher associated costs. For this reasons, the use of commercial off-the-shelf components (COTS), tested for their radiation hardness is nowadays a solution of choice, but this approach implies extensive testing for space operation qualification^1^.

Early research done by Capasso *et al*. some twenty years ago focused on new semiconductor coherent sources operating in the mid and far IR spectral range, the quantum cascade lasers – QCLs, opened new opportunities for spectroscopy at these wavelengths^5^. Such developments facilitated the spread of applications related to astronomy, astrophysics, astrochemistry, space missions^6^,^7^. High resolution spectroscopy of molecules rotation spectra is performed by SOFIA (Stratospheric Observatory for Infrared Astronomy) using heterodyne based on a QCL^8^. Other applications of QCLs for gas trace detection in planetary missions were planned^9^. The CubSat and ESA In-Orbit Demonstration unit were designed to accommodate a Laser Heterodyne Radiometer (LHR) using a QCL-based local oscillator^10^.

In the context implying the replacement of FTIR instrumentation designed for space missions by QCL-based equipments operating in the mid-IR, the core components involved into such setups (emitters, detectors, optical parts) have to be tested under irradiation environments.

Reports exist on the effects of various ionizing radiations on optical materials, such as:

(i) radiation induced absorption–RIA:

- observed over 200–800 nm for electron beam irradiated SiO_2_ at room temperature and heated^11^;

- in BaF2 cylindrical crystals, subjected to gamma radiation (^60^Co and ^137^Cs sources)^12^, or irradiated with various heavy ions^13^, having optical transmission measured in the 200–800 nm spectral range;

- over the 300 nm–850 nm spectral interval, in CaF_2_ crystals exposed to thermal and fast neutron flux^14^ or gamma-rays^15^;

- investigated in the visible-near IR (200 nm to 900 nm), in low OH content KUVI-S and high OH content KU-1 quartz glasses, and sapphire as they were subjected to electron beam, neutron, gamma-rays^16^,^17^,^18^,^19^,^20^;

- measured by FTIR in the IR (1600 cm^−1^ – 4000 cm^−1^) for neutron irradiated sapphire^21^.

(ii) radiation induced luminescence – RIL:

- signal in SiO_2_, KU-1 glass or sapphire under electron beam, gamma-ray, neutron proton or heavy ion irradiation^11^,^16^,^17^,^22^,^23^,^24^;

- appearing in BaF_2_ crystals during gamma^12^ or X-ray irradiation^25^;

- present in CaF_2_ crystals where they are exposed to X-rays^26^;

- emission in ZnSe under X-ray excitation^27^.

In the last 15 years, THz investigations of various materials gained momentum, as new techniques started to be used for dielectric materials identification/characterization in the far IR, through the evaluation of the dielectric constant and loss tangent by time-domain terahertz spectroscopy, which make possible the measurement of both the amplitude and phase of the detected signal, or by estimating the absorption coefficients and the refractive indices of various glasses and ceramics^28^,^29^,^30^,^31^,^32^,^33^,^34^,^35^,^36^.

More recently, the investigations of some active and passive optical components subjected to ionizing radiation were performed to evaluate their radiation resistivity^37^,^38^,^39^.

In this paper, we report the alpha particle irradiation induced changes in four mid-IR window materials (CaF_2_, BaF_2_, Zn Se, sapphire), for their possible use in spaceborne applications. For ***the first time***, the irradiation-induced characteristics are assessed by a multidisciplinary approach through optical investigations: UV to far-IR optical spectroscopy, radioluminescence measurements, THz spectroscopy and imaging.

## Results

*“Supplementary materials” contains information on:* (*i*) *additional results* (*section “Additional results”*); (*ii*) *the materials tested under this research and their specific characteristics of interest for this demarche* (*section ”Investigated samples”*); (*iii*) *the irradiation conditions* (*section “Alpha particle irradiation and simulations”*); (*iv*) *the laboratory setups used for the evaluation of irradiated sample* (*sections “Optical investigations”, “THz spectral investigations THz imaging”*)*. Figures and Tables to be found in this part of the work are cited in the main text accompanied by the suffix “Supplementary”.*

**Figure 1:**
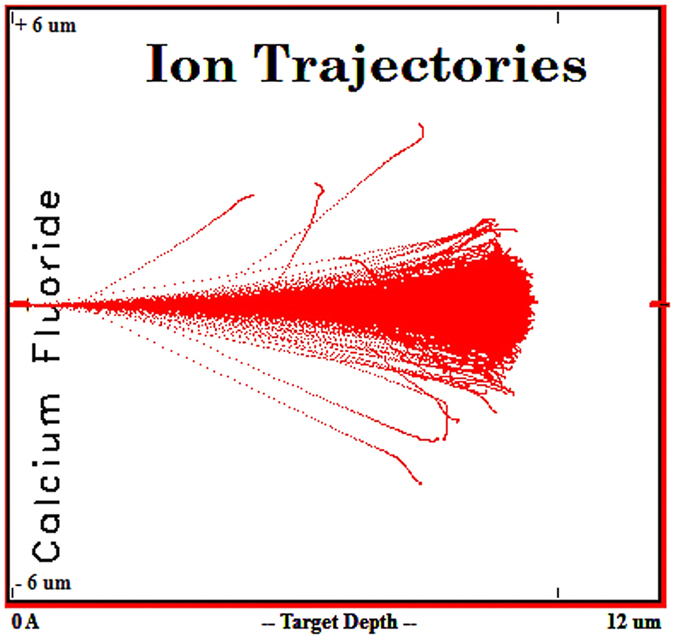
Result of the penetration depth simulation and scattering pattern of alpha particles in CaF_2_.

The simulation result referring to the penetration depth and scattering of alpha particles in CaF_2_ is shown in Fig. 1. The simulations for BaF_2_, Al_2_O_3_ and ZnSe are given in Supplementary Fig. 1, under “Additional results”. The simulations were done for beam charge of 100 000 alpha particles.

The penetration depths and the optical material mass affected by the exposure to alpha particles, as calculated according to equations Supplementary 1 and Supplementary 2, are depicted for comparison in Supplementary Fig. 2. The diagrams indicate that, on the first part of their trajectory, the particle path is quite straight as the particle energy is high, while, as particle interact with electrons, their energy diminish below the ionization threshold and it loses the velocity and produces nucleus displacements, which in turn contribute to material local density change^40^.

The material is described by the chemical bonds, density and molar mass that influences the probability of collisions of alpha particle with sample lattice [Supplementary 1–5], hence determining the number of produced vacancies (Supplementary Fig. 3). In the graphs, the number of collisions is plotted as a function of penetration depth.

The Rutherford backscattering spectrometry (RBS) measurements and the corresponding simulations were run in order to: (i) check samples’ compositions, (ii) identify impurities, (iii) estimate the charge deployed during the irradiation for the calculation of the total dose. The results of our experimental findings and simulations are compared in Supplementary Fig. 4.

**Figure 2:**
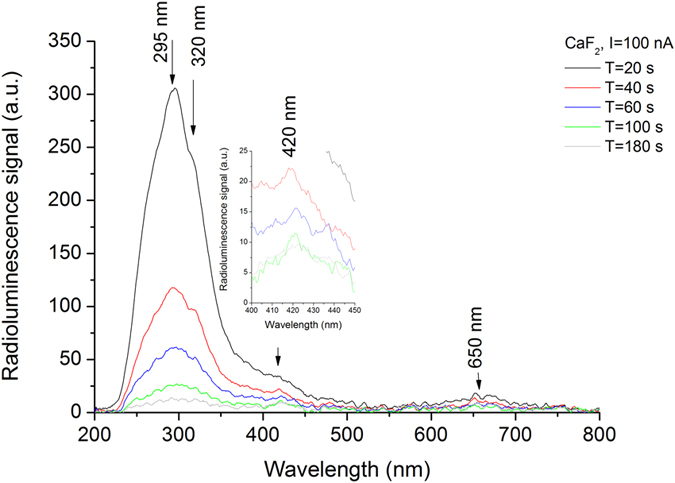
Radioluminescence detected spectrum under alpha particle irradiation of CaF_2_.

By on-line measurements we monitor the radioluminescence signal generated during the irradiation by alpha particles for the four mid-IR optical materials (Fig. 2 and Supplementary Fig. 5). In each case, the beam current is specified and the detected radioluminescence peaks are identified.

**Figure 3:**
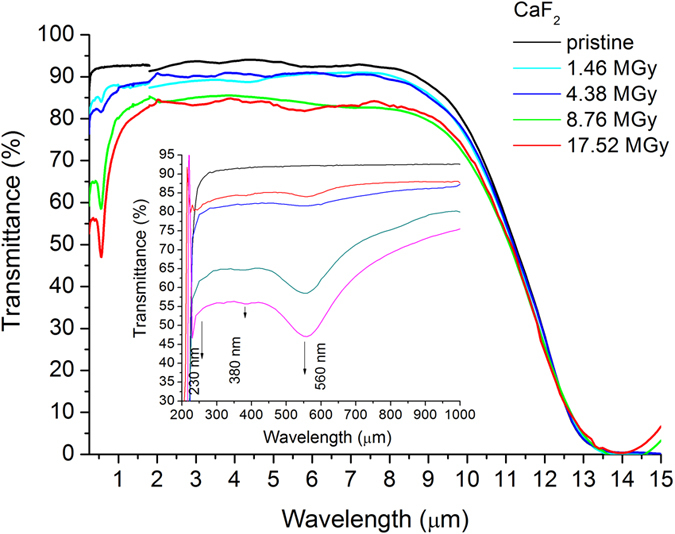
Spectral optical transmittance before and after alpha irradiation of CaF_2_.

Alpha particles incident on the optical windows materials produce color change as well as small craters or cracks, implying the degradation of the surface quality (Supplementary Fig. 6). This irradiation induced color modification is highlighted in Fig. 3 and Supplementary Fig. 7, as proved by the variation of the optical transmission. The details regarding the spectral absorbance are given in Supplementary Fig. 8 for easier identification of absorption peaks. The spectral diffused reflectance of the samples in the visible and near-IR spectral range is shown in Supplementary Fig. 9. The dose received by each sample by the subsequent irradiation sessions and the total dose are calculated according to Supplementary 1 – Supplementary 3 equations, in the *“Supplementary materials”* part of the paper.

**Figure 4:**
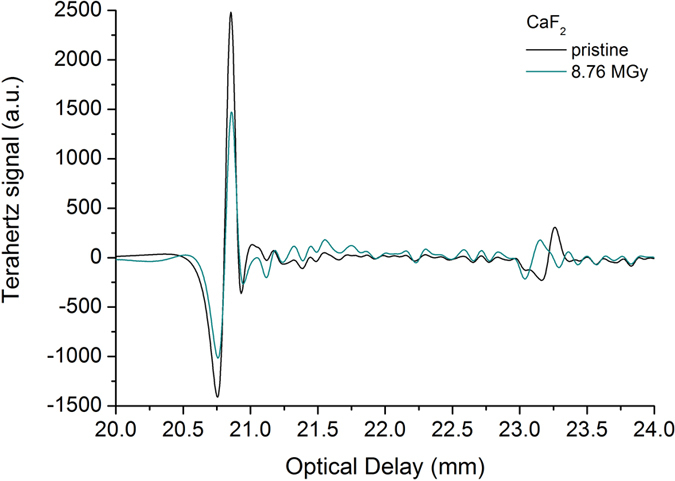
Delay of the transmitted THz pulse as induced by alpha particle irradiation for the case of CaF_2_.

One of the original points of our investigation is the study of the changes in the THz characteristics of the mid-IR materials, as induced by alpha particle irradiation. These modifications (i.e. density modifications) can be conveniently located if the THz pulse propagation is studied. THz techniques were used to evaluate crystallinity of the materials^41^,^42^. The optical delay of the THz transmitted signal for pristine and irradiated CaF_2_ samples is given in Fig. 4, while the optical delay of the THz reflected signal for pristine and irradiated BaF_2_, sapphire and ZnSe samples is given in Supplementary Fig. 10.

The THz signal, as obtained in the frequency domain, for the tested samples prior and after the irradiation is represented in Supplementary Fig. 11. The THz spectroscopy was proposed for the measurements of optical characteristics (refractive index, absorbance)^43^,^44^ and real/imaginary part of the dielectric constant^44^,^45^ of various materials. The results of our investigation for the mid-IR windows, as they are affected by the irradiation, are depicted in Supplementary Figs 12–15.

**Figure 5:**
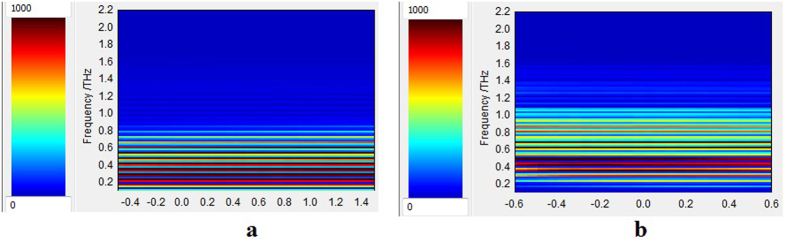
THz reflectance 2D image along the *x* axis as function of THz frequency (B-scan) using the frequency domain analysis mode for CaF_2_ sample: (**a)** pristine window; (**b**) window exposed up to the dose of 8.76 MGy.

To further illustrate the use of THz methods in investigating the effects of alpha particle irradiation on mid-IR materials we provide some examples of THz imaging studies for pristine and irradiated CaF_2_; BaF_2_; Al_2_O_3_ and ZnSe samples. The variation of the THz reflected signal as a function of THz frequency for the pristine and exposed windows, as found by the frequency domain analysis mode, is shown in Fig. 5 and Supplementary Fig. 16. The images illustrate the variation along the *x* axis (B-scan). For the detection geometry of the reflected signal, please refer to *“THz spectral investigations THz imaging”* section of the *Supplementary materials*. The images depicted in Fig. 5 and Supplementary Fig. 16 correspond to the variation of the THz signal with frequency, as represented in Supplementary Fig. 11.

**Figure 6:**
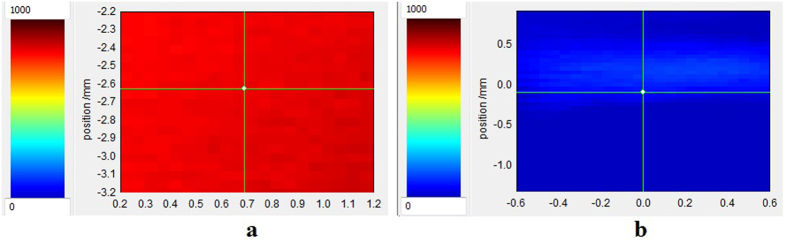
THz cross section in the *xy* plane (C-scan) using the frequency domain analysis mode for CaF_2_ sample: (**a**) pristine window; (**b**) window exposed up to the dose of 4.38 MGy.

The examples of *xy* cross sections (C-scan) through the tested samples, as generated by THz reflectance imaging before and after the irradiation, are given in Fig. 6 and Supplementary Fig. 17. Figure 6 illustrates the irradiation induced change detected under THz radiation in the CaF_2_ sample over an area having no structural defects. Similar data are provided for Al_2_O_3_ (Supplementary Fig. 17c and d) and ZnSe (Supplementary Fig. 17e and f). In Supplementary Fig. 17a and b, the THz image of a defect localized in the BaF_2_ sample is shown before and after the exposure.

The detection of the defect through THz signal reflection, as represented in the frequency domain analysis mode, can be seen very well in Supplementary Fig. 18. This information corresponds to the results presented in Supplementary Fig. 17a and b.

The similar results to the optical delay signal (Fig. 4 and Supplementary Fig. 10), represented as 2D images picked up along the *Ox* axis and using the time domain analysis, are shown for pristine and irradiated windows in Supplementary Fig. 19.

## Discussions

From the simulations run it can be noticed that the alpha particles scatter pattern and the penetration depth (Fig. 1 and Supplementary Fig. 1) are dependent on the sample material (i.e. density, molar mass). The collision events which might produce vacancies occur at specific depths in CaF_2_, Al_2_O_3_ or ZnSe and they spread over the entire interaction length in BaF_2_ (Supplementary Fig. 3).

The behavior of the tested samples under irradiation can be influenced by some impurities. Within this context we performed RBS investigations (Supplementary Fig. 4). No impurities were detected in our measurements having the sensitivity of about 1%.

As for CaF_2_, the main radioluminescence peak is observed in the UV range (Fig. 2) with components at λ = 295 nm, 320 nm and 420 nm^26^,^46^ and a much smaller component is detected in visible (λ = 650 nm), as it was found in the earlier published reports.

Under alpha particle exposure, BaF_2_ exhibits a broad band emission from 250 nm to 750 nm (Supplementary Fig. 5a), where some peaks or spectral bands previously reported in the literature can be identified^25^,^47^.

The radioluminescence from irradiated sapphire presents several peaks in UV (λ = 280 nm^24^, 325 nm^17^, 420 nm^17^,^18^,^22^,^23^,^24^), one major in the near-IR (λ = 697 nm^17^,^18^,^24^) and some smaller peaks in the same region^24^. In the case of ZnSe, the emitted spectra are present for λ = 473 nm^27^. These peaks for sapphire and ZnSe were identified also in our study (Supplementary Fig. 5b and c).

Regarding the radiation induced optical attenuation we have noticed that:

- CaF_2_ decreases its optical transmission (Fig. 3) from 350 nm to 11 μm with a significant deep in UV-visible (up to 1 μm);
- BaF_2_ transmission (Supplementary Fig. 7a) decreases in the UV (below 500 nm) and mid-IR ranges;
- sapphire is almost not affected by the exposure (Supplementary Fig. 7b), excepting some small attenuation pecks in the near-IR (0.9 μm–1 μm) and the region below 300 nm;
- ZnSe transmission (Supplementary Fig. 7c) is diminished over the entire operating range (530 nm–19 μm);
- The absorption peaks related to the radiation effects in the studied materials were identified as follows:
- in CaF_2_ (Supplementary Fig. 8a) at λ = 379 nm^14^ and 550 nm^13^,^14^;
- in BaF_2_ (Supplementary Fig. 8b) at λ = 220 nm, 240 nm, 750 nm^13^;
- in sapphire (Supplementary Fig. 8c) at λ = 220 nm^18^,^22^,^29^, 257 nm^18^,^19^,^20^,^22^, 320 nm^18^.

Three of the tested samples (CaF_2_, BaF_2_, Al_2_O_3_) are purchased from the same manufacturer, while the ZnSe sample was acquired from another provider. As the materials are exposed to incident particles their diffused reflectance increases (Supplementary Fig. 9), supposedly as function of their mean harness (Supplementary Table 1). The diffused reflectance increase can be attributed to the surface defects (small craters/cracks) produced by the irradiation (Supplementary Fig. 6).

As the samples are subjected to alpha particle bombardment, their local properties changes were detected as a delay present in the propagation of the THz investigation pulse (Fig. 4 and Supplementary Fig. 10). Within some limits, 27.3 × 10^6^ Gy for BaF_2_, 8.67 × 10^6^ Gy for CaF_2_, alpha particle irradiation produces a change of the THz frequency signal, accompanied by a shift of the maximum towards higher frequencies (Supplementary Fig. 11a and b), while in the case of Al_2_O_3_ and ZnSe the THz frequency spectrum does not change and only a decrease of the amplitude can be noticed (Supplementary Fig. 11c and d). At higher total doses for CaF_2_ and BaF_2_ the frequency shift saturates.

Some modifications of the THz spectral refractive index upon irradiation occur for the sapphire and CaF_2_ samples, while ZnSe and BaF_2_ refractive index variation is smaller (Supplementary Fig. 12). The THz absorbance of CaF_2_, BaF_2_ seems not to be affected by the irradiation (Supplementary Fig. 13a and b), and some changes being observed for Al_2_O_3_ and ZnSe (Supplementary Fig. 13c and d).

The exposure to alpha particles produces a variation of the real part of dielectric constant in the case of all tested samples, while the imaginary part remains almost unchanged (Supplementary Figs 14 and 15).

The capability to monitor the irradiation induced changes by THz spectroscopy is provided by 3D mapping of these effects in the irradiated volume using different cross sections, as it is demonstrated through THz reflectance measurements (Figs 5 and 6, Supplementary Fig. 16 and Supplementary Fig. 17). The THz imaging can be used to localize material defects or anisotropic points (Supplementary Fig. 18). Additional interpretations can be derived from images obtained by time domain analysis (Supplementary Fig. 19).

After the irradiation the CaF_2_ and BaF_2_ samples became very brittle, as that they presented cracks or even at the end were broken into several pieces. This outcome can be attributed to the lower thermal conductivity of CaF_2_ and BaF_2_, as the irradiations took place in vacuum, hence poorer heat dissipation occurred in these samples producing local thermal stress.

## Conclusions

We performed our study by combining various techniques in order to assess for the ***first time*** the changes induced in mid-IR optical materials by alpha particle irradiation. Our investigations bring several novelties to the field of material research operating in harsh environments (i.e. ionizing radiation):

1. We evaluate, as a premiere, the modifications produced by alpha particles in IR optical windows as it concerns: (i) the optical transmission over their entire spectral operating range of these materials (Fig. 3 and Supplementary Fig. 7 and Supplementary Fig. 8); (ii) the degradation of their surface roughness post irradiation (spectral diffusion reflectance measurements, Supplementary Fig. 9); (iii) the generated radioluminescence signal (Fig. 2 and Supplementary Fig. 5).
2. We provide information on the interaction between the target material and the alpha particle beam, supported by the simulations run in relation to the penetration depth, the particle scattering inside the sample and the number of generated vacancies which might be responsible for the optical transmission degradation (Fig. 1, Supplementary Fig. 1, Supplementary Fig. 3).
3. We offer data, not available up today, on the THz refractive index, absorbance, real and imaginary parts of the dielectric constant for four mid-IR materials prior and post exposure to alpha particles (Supplementary Figs 11–14).
4. For the first time we highlight the change of these materials THz spectra, as they are subjected to irradiation (Fig. 5, Supplementary Fig. 11 and Supplementary Fig. 16).
5. The effect of the irradiation on mid-IR materials THz performances are also exemplified by the modification THz pulse delay (Fig. 4 and Supplementary Fig. 10).
6. We propose THz imaging as a mean to “visualize” the irradiation produced changes in the volume of the irradiated material, along different axes (Figs 5 and 6, Supplementary Figs 16–19).

The results of this research, constituted into an extensive database, are of interest for researchers involved in: (i) material science studies, (ii) investigation of ionizing radiation impact on optical materials, (iii) applications of THz technology, (iv) the study of scintillating materials, (v) the design of equipments to operate in harsh environments (i.e. spaceborne instrumentation).

## Materials and Methods

The mid-IR optical windows tested under this investigation were provided by Skight Optics Co., Ltd., China (CaF_2_, BaF_2_, Al_2_O_3_) and Edmund Optics Ltd. (ZnSe). General characteristics of the studied materials are published in literature [Supplementary 1–5]. Specific parameters of the samples (dimensions and operating spectral range) are indicated in Supplementary Table 1 (*“Supplementary materials”,* section “*Investigated samples”*).

Irradiation conditions are described *“Supplementary materials”,* under section “*Alpha particle irradiation and simulations*”.

The irradiations were run in several subsequent steps, and, before and after each irradiation, their characteristics were measured in the optical and THz spectral range. For details concerning the setups use in laboratory (off-line) optical and THz investigations refer to *“Supplementary materials”,* sections “*Optical investigations*” and *“THz spectral investigations THz imaging”*.

## Additional Information

**How to cite this article:** Sporea, D. *et al*. Optical and THz investigations of mid-IR materials exposed to alpha particle irradiation. *Sci. Rep.* **7**, 40209; doi: 10.1038/srep40209 (2017).

**Publisher's note:** Springer Nature remains neutral with regard to jurisdictional claims in published maps and institutional affiliations.

## Supplementary Material

Supplementary Information
