# Supplementary material for: Optical and THz investigations of mid-IR materials exposed to alpha particle irradiation
Source: Sci Rep. 2017 Jan 9;7:40209. doi: 10.1038/srep40209 (PMC5220343; doi:10.1038/srep40209)
Supplement: Supplementary Information [file srep40209-s1.pdf]

Optical and THz investigations of mid-IR materials exposed to alpha particle irradiation

Dan Sporea[1*], Laura Mihai[1], Adelina Sporea[1], Ion Vâţã[2]

[1]National Institute for Laser, Plasma and Radiation Physics, Center for Advanced Laser Technologies, Magurele, RO-077125, Romania

[2]"Horia Hulubei" National Institute of Physics and Nuclear Engineering, RO-077125, Magurele, Romania

[*]Corresponding author: dan.sporea@inflpr.ro

Supplementary information

Additional results

Supplementary Fig. 1 reproduces the simulation of alpha particle penetration depths and scattering patterns in BaF_2, Al_2O_3, ZnSe.

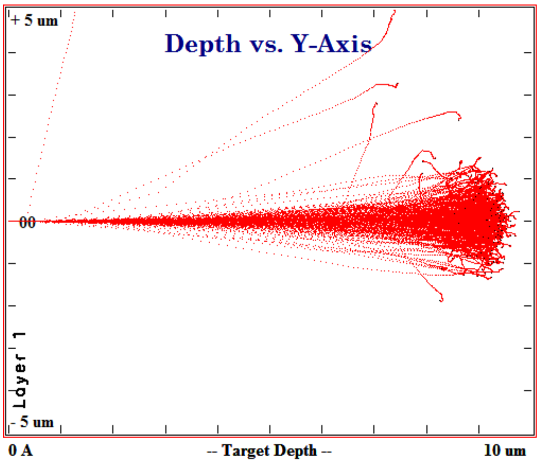

a

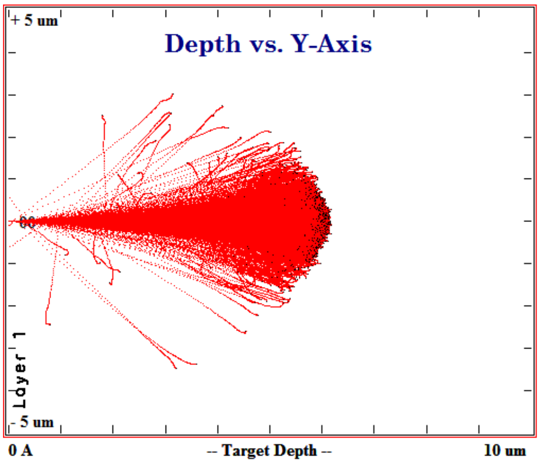

b

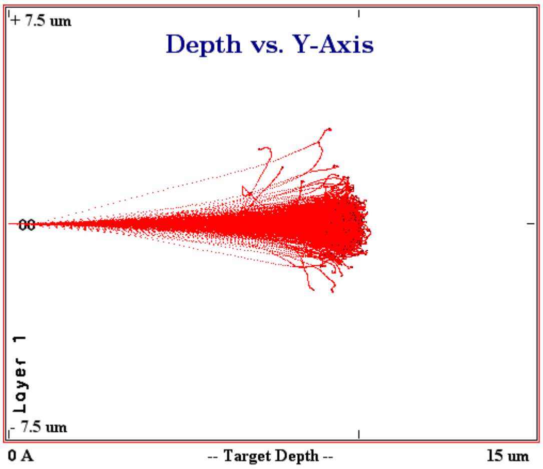

c

Supplementary Figure 1. Results of the penetration depth simulation and scattering pattern of alpha particles in: a – BaF_2; b – sapphire; c – ZnSe.

In Supplementary Fig. 2 are given the penetration depth and the sample mass affected by alpha particle irradiation as resulted from the simulations.

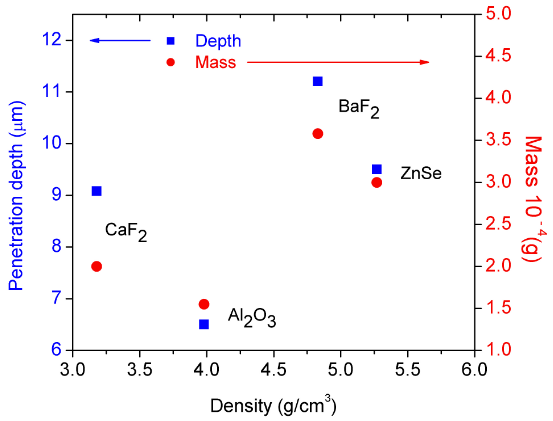

Supplementary Figure 2. Penetration depth and the optical material mass affected by alpha particle irradiation for the four windows, simulations done for a beam charge of 100 μC.

The results of the simulations regarding the number of vacancies produce by alpha particle incidence on each tested sample are provided in Supplementary Fig. 3.

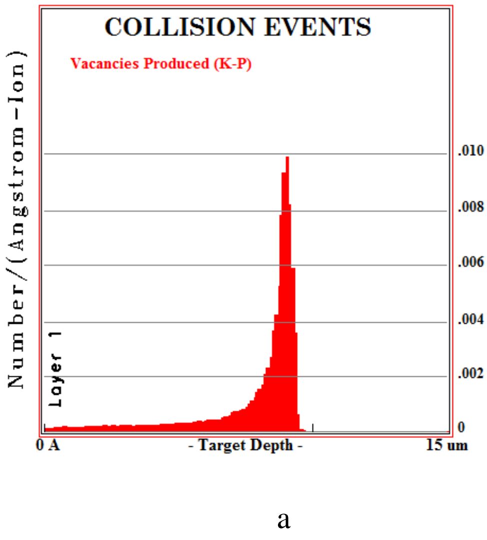

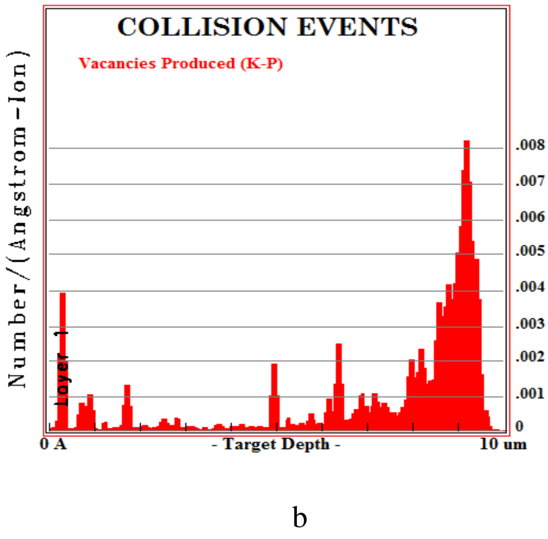

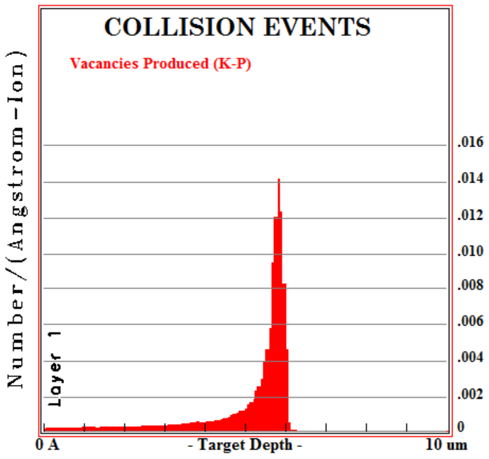

c

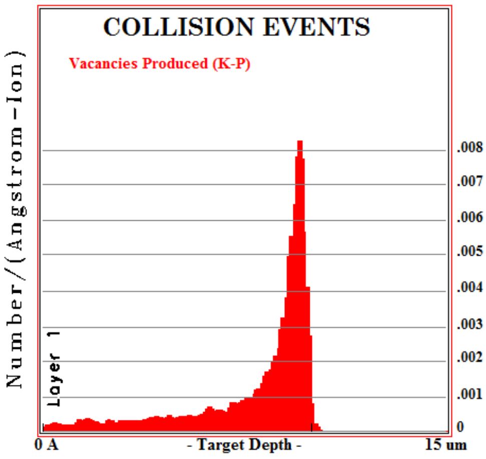

d

Supplementary Figure 3. Estimated number of vacancies produced upon alpha particles irradiation of: a – CaF_2; b – BaF_2; c – sapphire; d – ZnSe, as indicated by the simulations, function of the penetration depth.

Supplementary Fig. 4 illustrates the comparison between the simulated and measured RBS data.

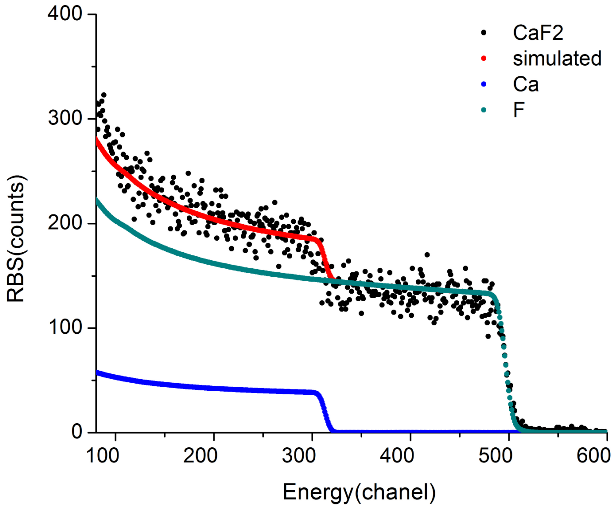

a

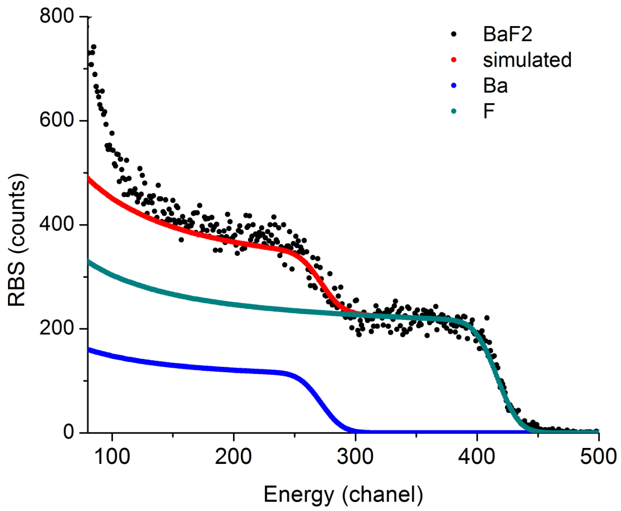
800
600
400
200
0
100
200
300
400
500
RBS (counts)
Energy (chanel)
BaF2
simulated
Ba
F

b

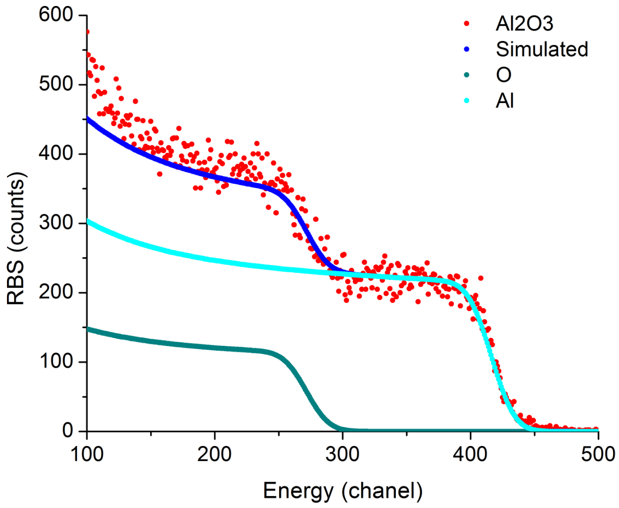
600
500
400
300
200
100
0
100
200
300
400
500
RBS (counts)
Energy (chanel)
Al2O3
Simulated
O
Al

c

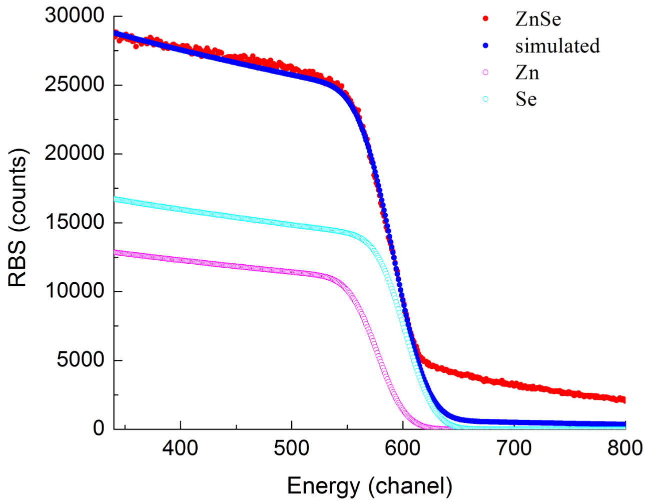

d

Supplementary Figure 4. Results of RBS measurements and simulations for: a – CaF_2; b – BaF_2; c – sapphire; d – ZnSe.

In Supplementary Fig. 5 the detected radioluminescence peaks are highlighted for: a – BaF_2, b – Al_2O_3 and c – ZnSe.

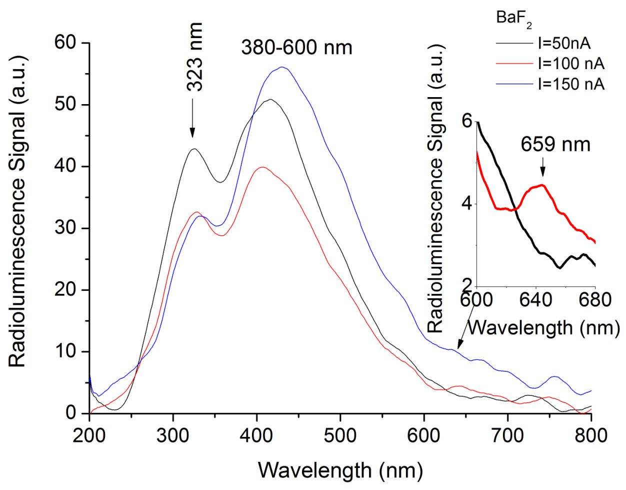

a

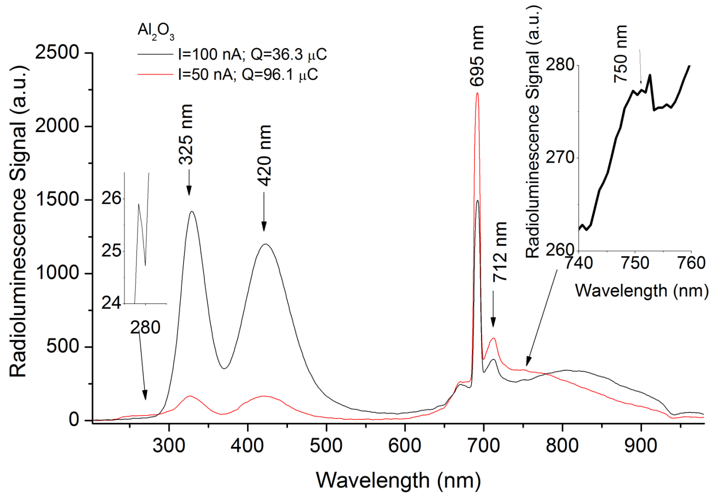

b

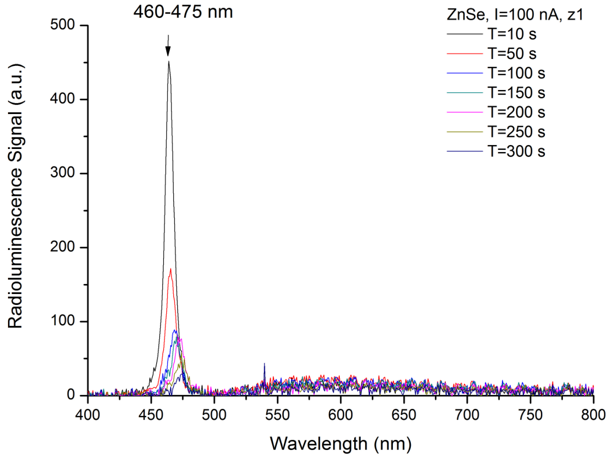

c

Supplementary Figure 5. Radioluminescence detected spectra under alpha particle irradiation of: a – BaF_2; b – sapphire; c – ZnSe.

The surface defects and color change induced by particle bombardment are detailed in Supplementary Fig. 6.

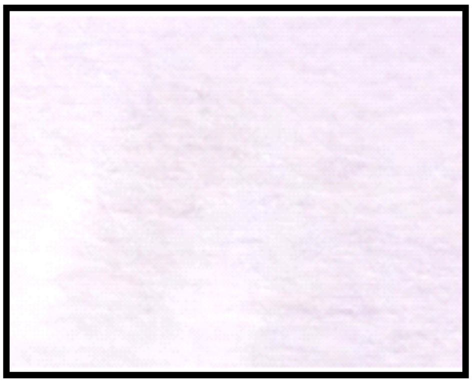

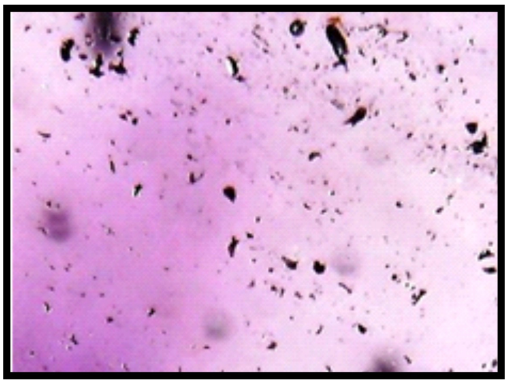

a

b

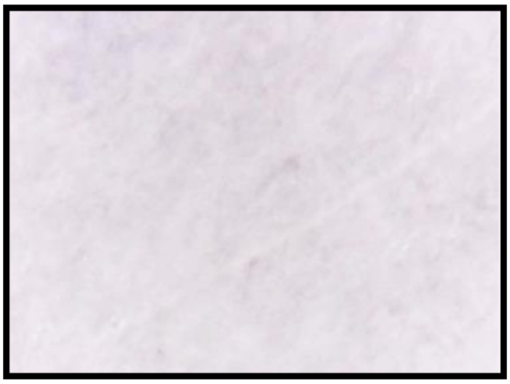

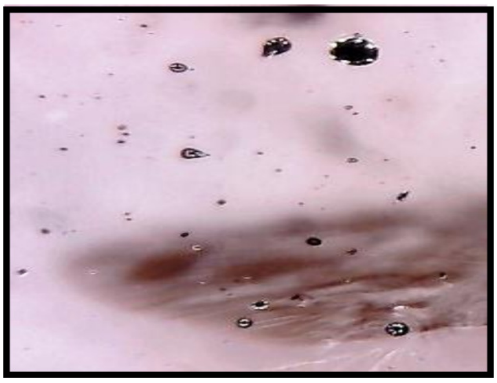

c

d

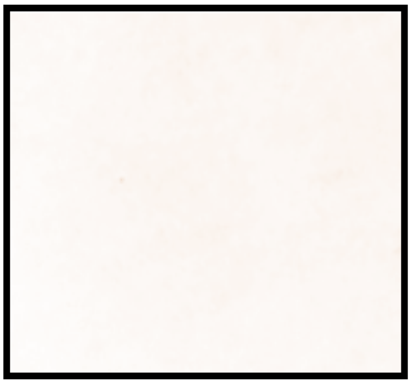

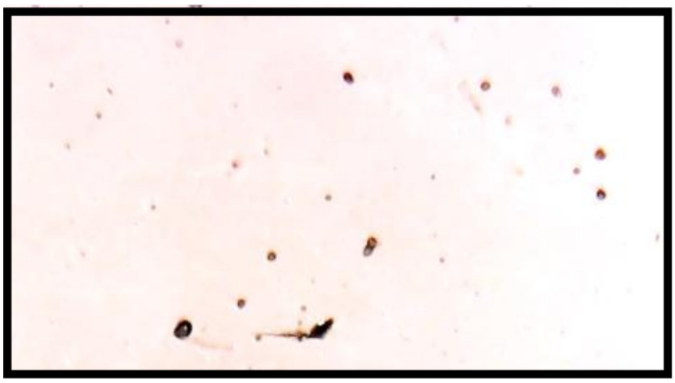

e

f

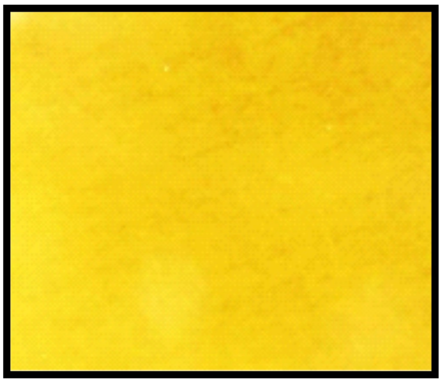

g

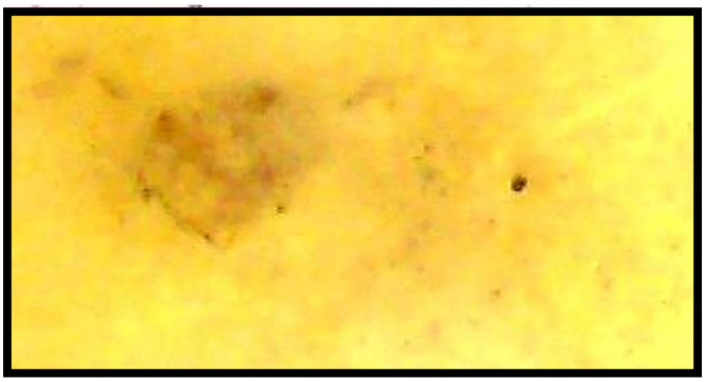

h

Supplementary Figure 6. Color change and development of surface defects in the alpha particle irradiated mid-IR optical materials: a – CaF_2 before irradiation; b – CaF_2 after irradiation (total dose 17.52 MGy); c – BaF_2 before irradiation; d – BaF_2 after irradiation (total dose 109.2 MGy); e – sapphire before irradiation (total dose 23.16 MGy); f – sapphire after irradiation; g – ZnSe before irradiation; h – ZnSe after irradiation (total dose 12 MGy).

The variation of the optical spectral transmittance of the samples before and after irradiation is illustrated in Supplementary Fig. 7.

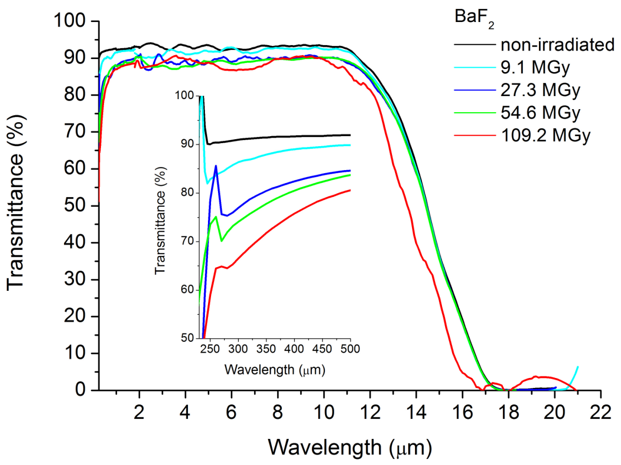

a

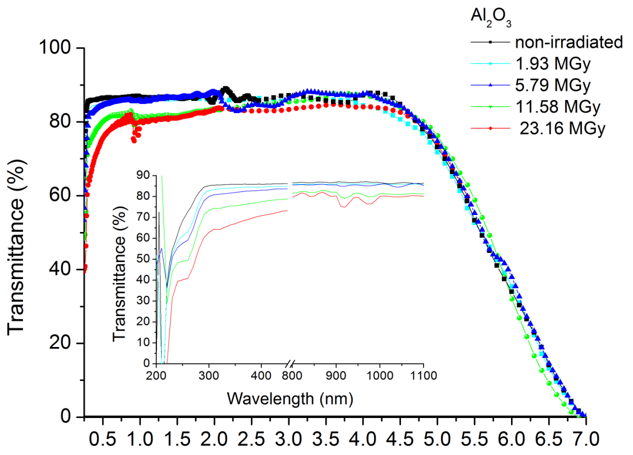

b

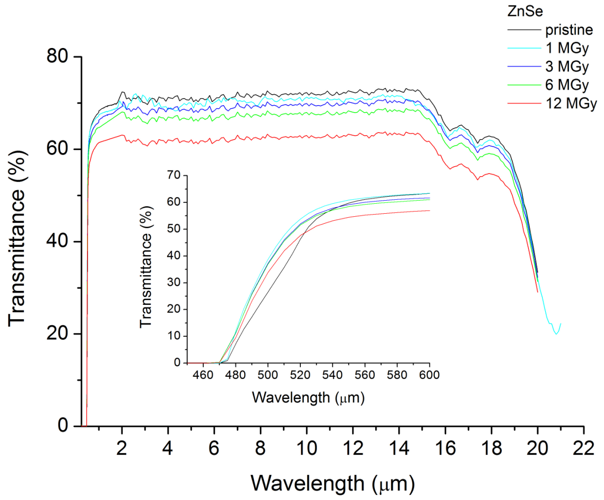

c

Supplementary Figure 7. Optical spectral transmittance before and after alpha irradiation of: a – BaF_2; b – sapphire; c – ZnSe.

For a better localization of the irradiation induced optical attenuation details of the optical spectral absorbance are indicated in Supplementary Fig. 8.

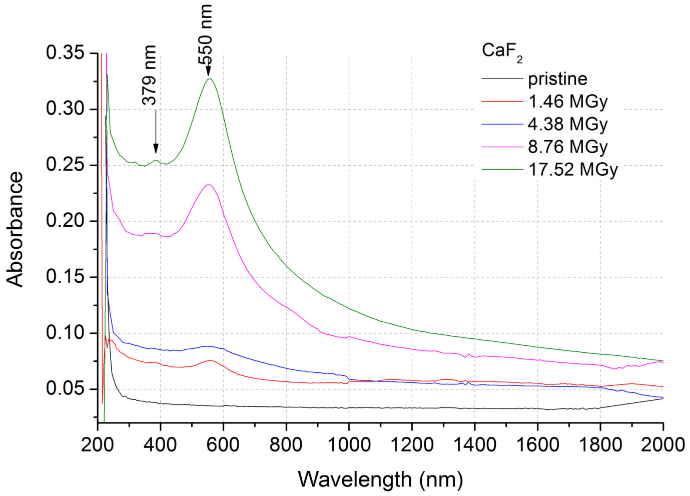
CaF2
pristine
1.46 MGy
4.38 MGy
8.76 MGy
17.52 MGy
379 nm
550 nm
Absorbance
Wavelength (nm)
0.35
0.30
0.25
0.20
0.15
0.10
0.05
200
400
600
800
1000
1200
1400
1600
1800
2000

a

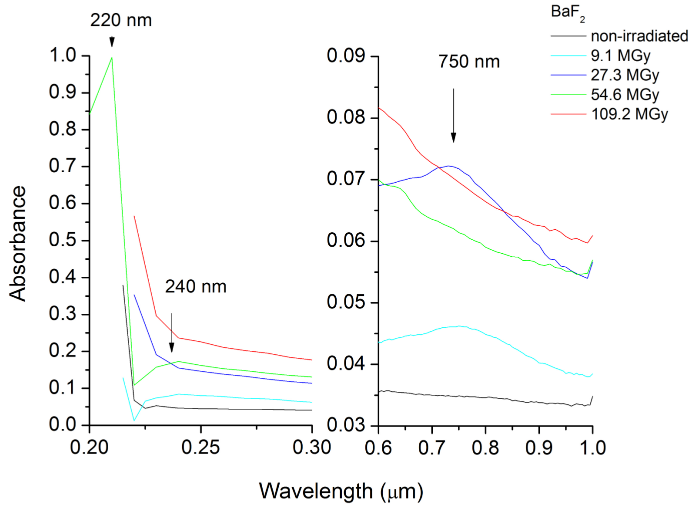
BaF2
non-irradiated
9.1 MGy
27.3 MGy
54.6 MGy
109.2 MGy
220 nm
240 nm
750 nm
Absorbance
Wavelength (μm)
1.0
0.9
0.8
0.7
0.6
0.5
0.4
0.3
0.2
0.1
0.0
0.20
0.25
0.30
0.09
0.08
0.07
0.06
0.05
0.04
0.03
0.6
0.7
0.8
0.9
1.0

b

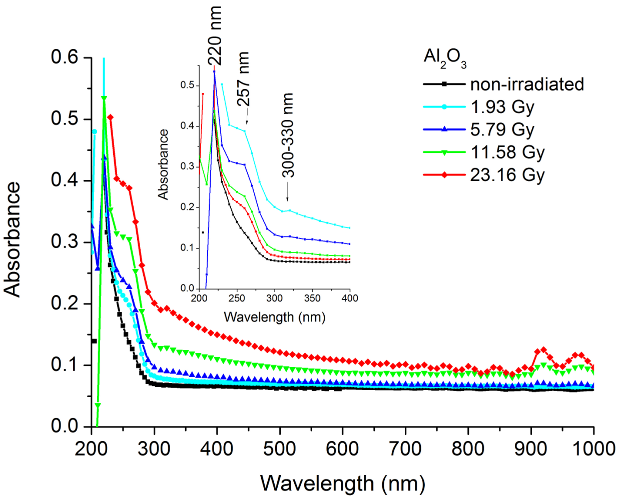

c

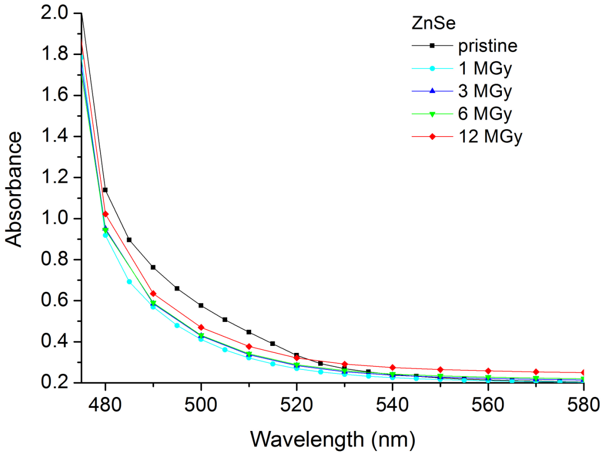

d

Supplementary Figure 8. Details of the optical spectral absorbance before and after subsequent irradiation steps for: a – CaF_2; b – BaF_2; c – sapphire; d – ZnSe.

The degradation of samples' surface quality under alpha particle irradiation can be estimated from the optical spectral diffused reflectance measurements (Supplementary Fig. 9).

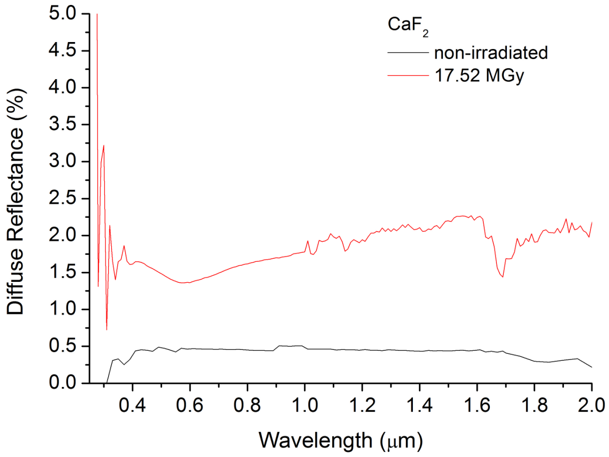
CaF2
non-irradiated
17.52 MGy
Diffuse Reflectance (%)
Wavelength (μm)
5.0
4.5
4.0
3.5
3.0
2.5
2.0
1.5
1.0
0.5
0.0
0.4
0.6
0.8
1.0
1.2
1.4
1.6
1.8
2.0

a

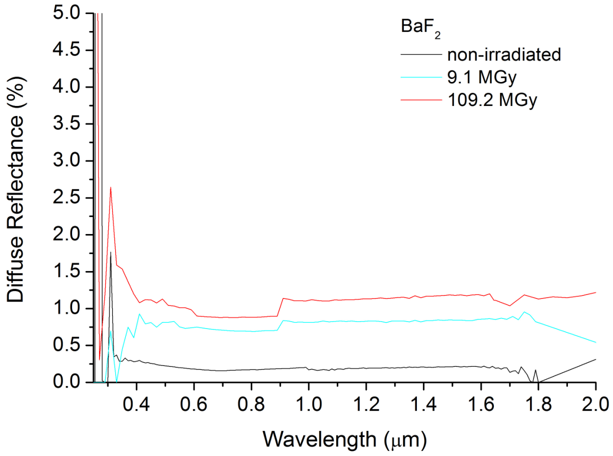
BaF2
non-irradiated
9.1 MGy
109.2 MGy
Diffuse Reflectance (%)
Wavelength (μm)
5.0
4.5
4.0
3.5
3.0
2.5
2.0
1.5
1.0
0.5
0.0
0.4
0.6
0.8
1.0
1.2
1.4
1.6
1.8
2.0

b

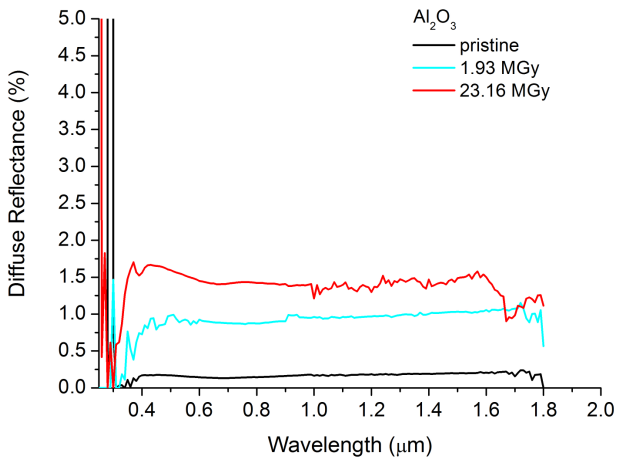

c

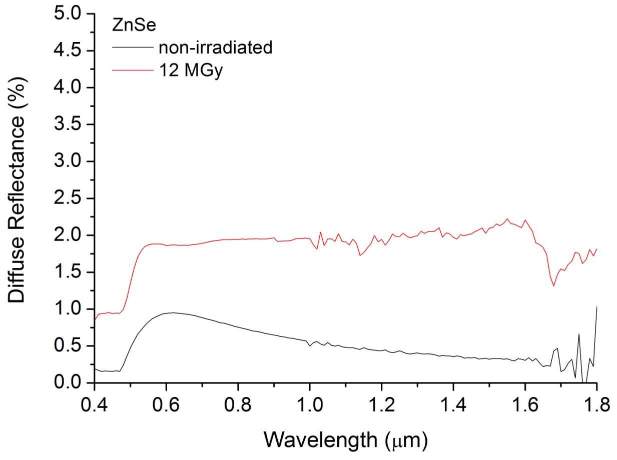

d

Supplementary Figure 9. Optical spectral diffused reflectance before and after alpha irradiation of: a – CaF_2; b – BaF_2; c – sapphire; d – ZnSe.

The effect of irradiation on investigated samples is illustrated in Supplementary Fig. 10, where the delayed pulse of reflected THz signal is shifted as function of the irradiation dose.

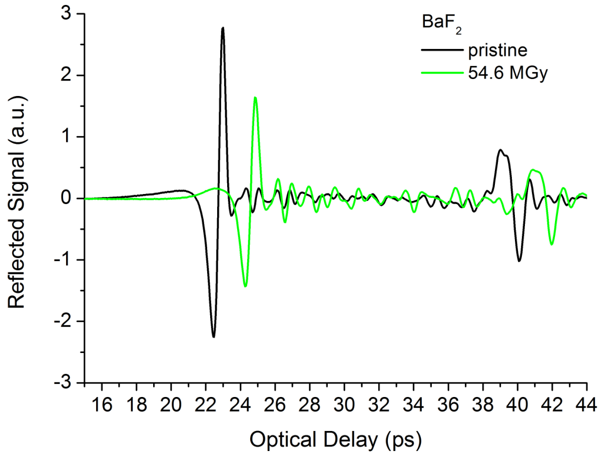
BaF2
pristine
54.6 MGy
Reflected Signal (a.u.)
Optical Delay (ps)

a

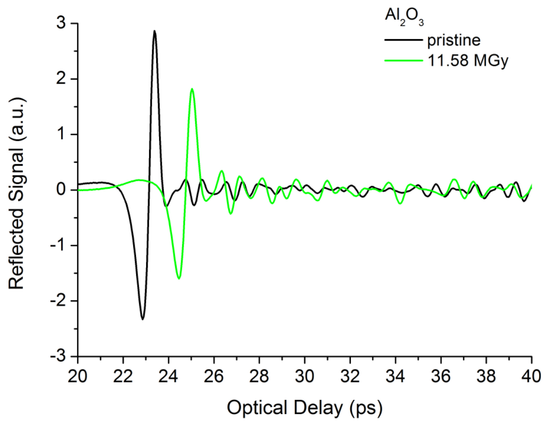
Al2O3
pristine
11.58 MGy
Reflected Signal (a.u.)
Optical Delay (ps)

b

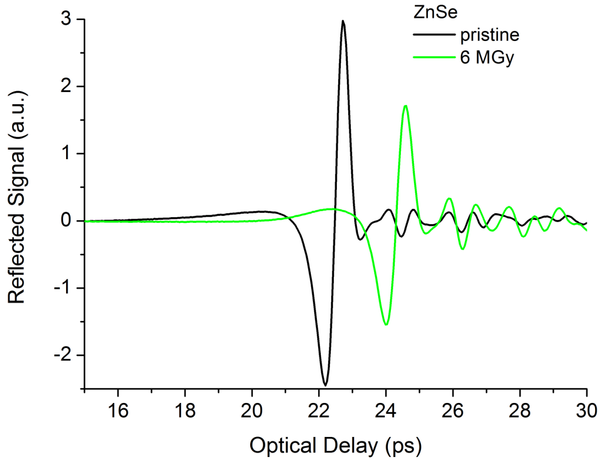

c

Supplementary Figure 10. THz pulse delay induced by alpha particle irradiation for: a – BaF_2; b – sapphire; c – ZnSe.

The reflected THz signal variation in frequency domain as it is changed by alpha particle irradiation is illustrated in Supplementary Fig. 11. The ration of the two signals is given in the inset. For readers' convenience, the water related absorption bands were removed.

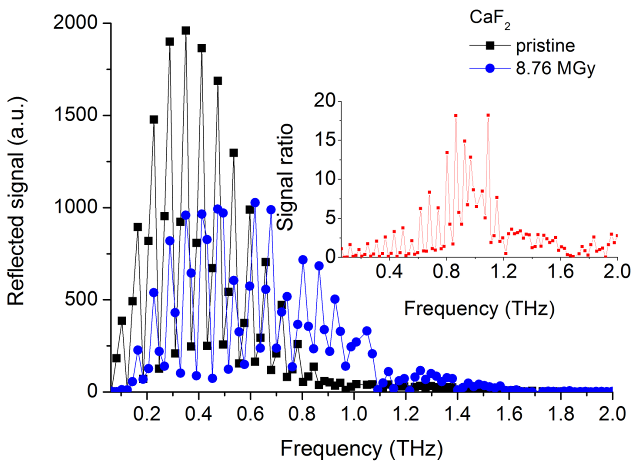

a

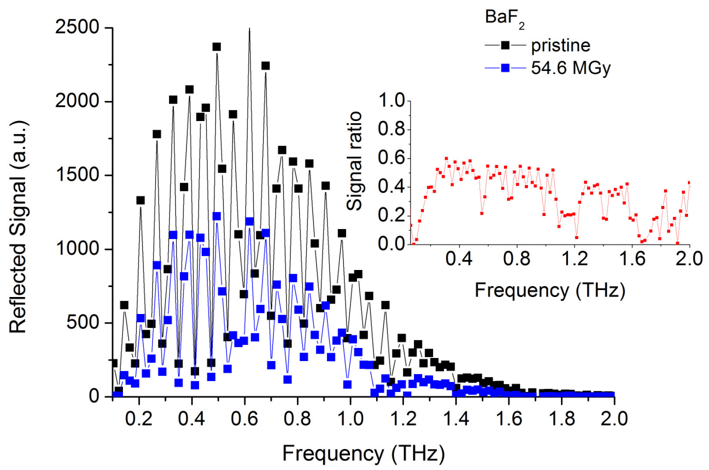
BaF2
pristine
54.6 MGy
Reflected Signal (a.u.)
Frequency (THz)
Signal ratio
Frequency (THz)

b

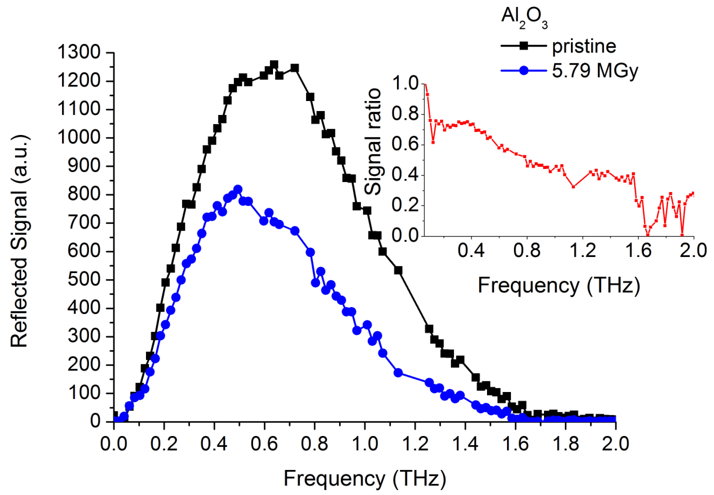
Al2O3
pristine
5.79 MGy
Reflected Signal (a.u.)
Frequency (THz)
Signal ratio
Frequency (THz)

c

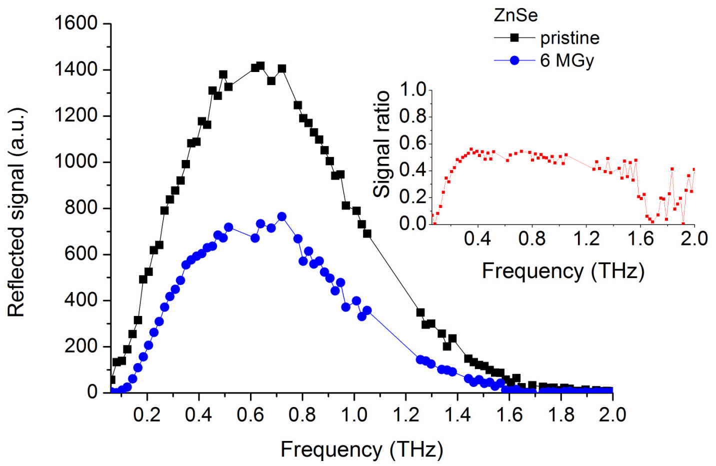

d

Supplementary Figure 11. Change of the THz spectra as affected by alpha particle irradiation in the case of: a – CaF_2; b – BaF_2; c – Al_2O_3; d – ZnSe.

The effects of alpha particle irradiation on the refractive index (Supplementary Fig. 12), absorbance (Supplementary Fig. 13), real part of the dielectric constant (Supplementary Fig. 14), and imaginary part of the dielectric constant (Supplementary Fig. 15) are depicted below.

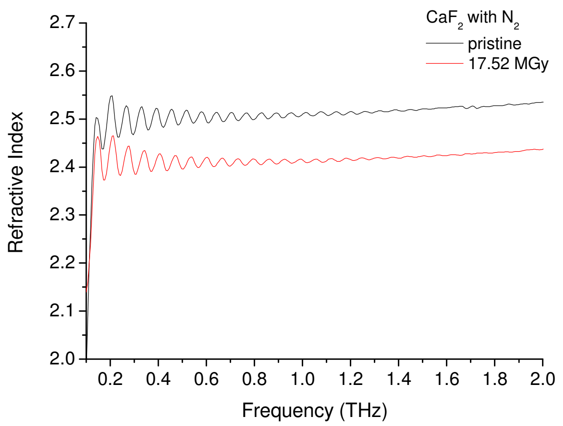

a

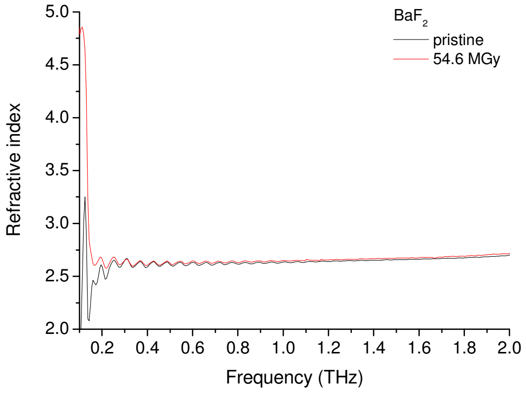
BaF$_2$
pristine
54.6 MGy
Refractive index
Frequency (THz)

b

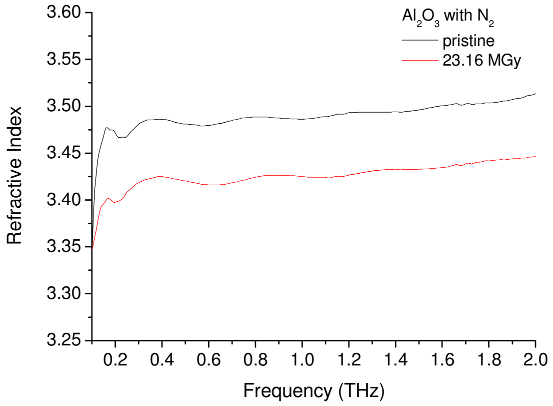
Al$_2$O$_3$ with N$_2$
pristine
23.16 MGy
Refractive Index
Frequency (THz)

c

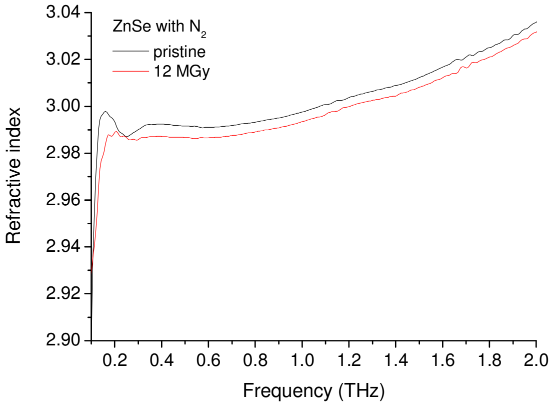
ZnSe with N$_2$
pristine
12 MGy
Refractive index
Frequency (THz)

d

Supplementary Figure 12. Refractive index change in the THz spectral range of irradiated: a – CaF_2; b – BaF_2; c – sapphire; d – ZnSe.

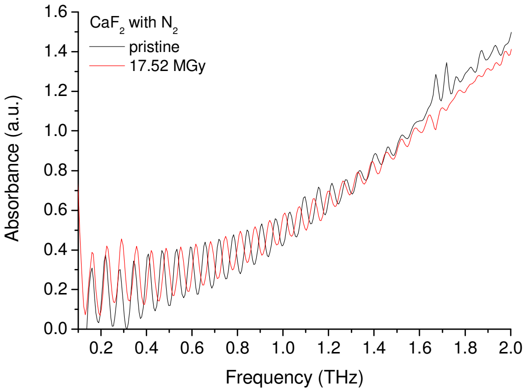

a

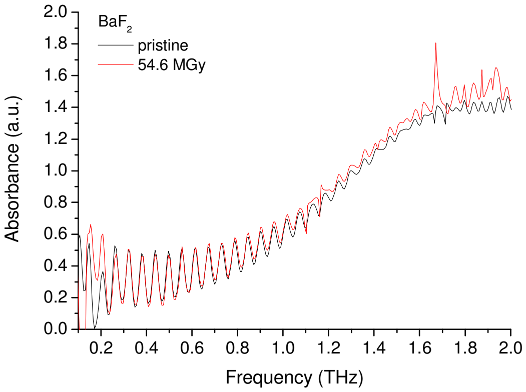

b

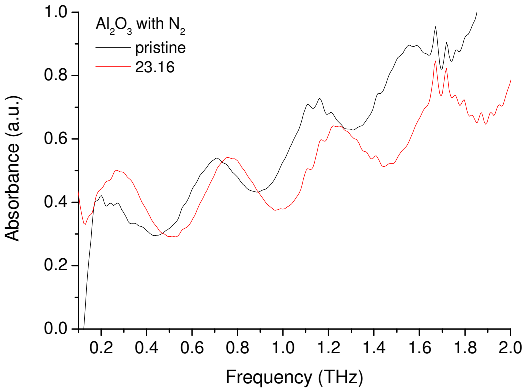

c

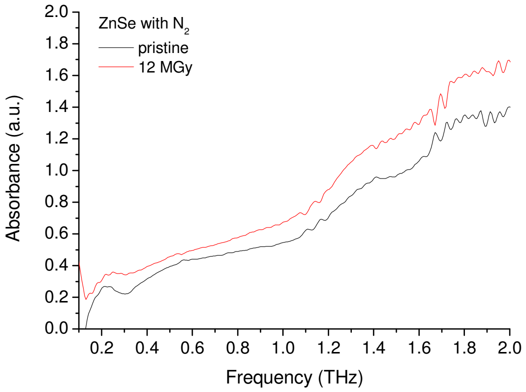

d

Supplementary Figure 13. Absorbance change in the THz spectral range of irradiated: a – CaF_2; b – BaF_2; c – sapphire; d – ZnSe.

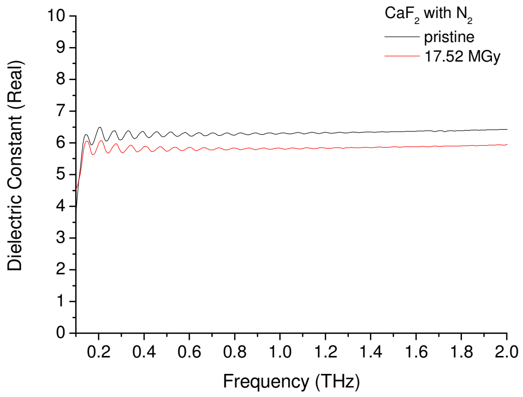
CaF2 with N2
pristine
17.52 MGy
Dielectric Constant (Real)
Frequency (THz)

a

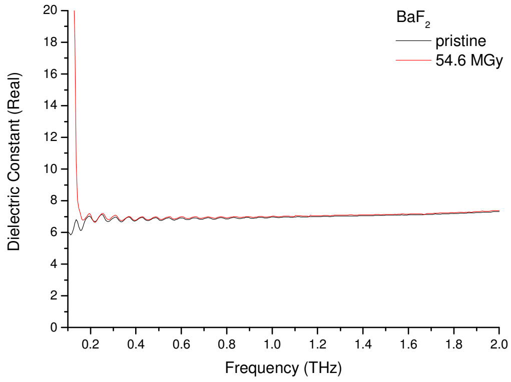
BaF2
pristine
54.6 MGy
Dielectric Constant (Real)
Frequency (THz)

b

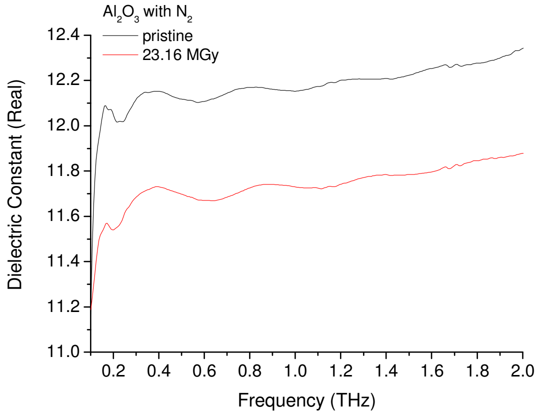
Al2O3 with N2
pristine
23.16 MGy
Dielectric Constant (Real)
Frequency (THz)

c

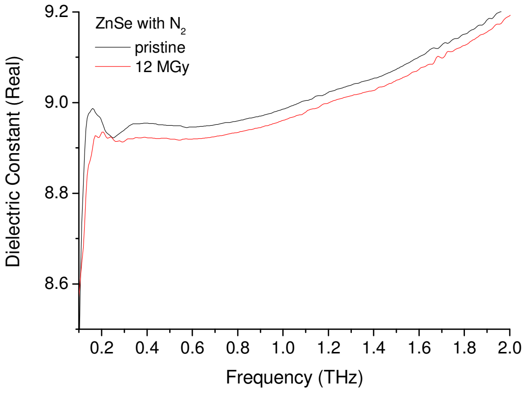

d

Supplementary Figure 14. Irradiation induced changes of the dielectric constant real part for: a – CaF_2; b – BaF_2; c – sapphire; d – ZnSe.

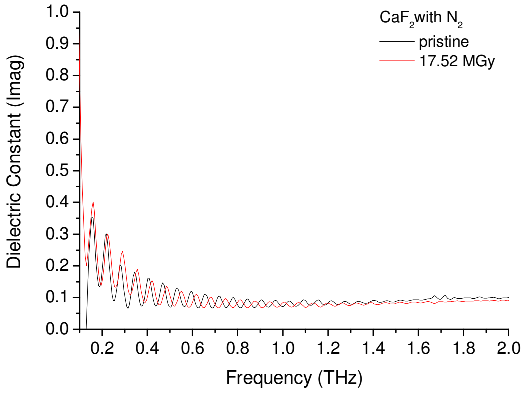

a

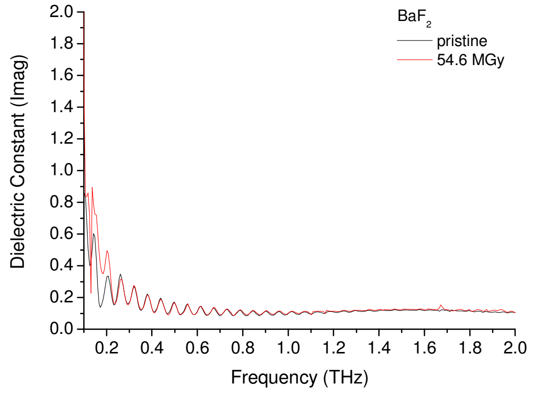

2.0
1.8
1.6
1.4
1.2
1.0
0.8
0.6
0.4
0.2
0.0
Dielectric Constant (Imag)
0.2
0.4
0.6
0.8
1.0
1.2
1.4
1.6
1.8
2.0
Frequency (THz)
BaF_2
pristine
54.6 MGy

b

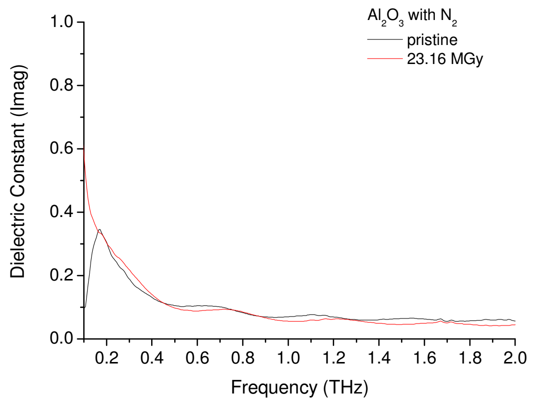

1.0
0.8
0.6
0.4
0.2
0.0
Dielectric Constant (Imag)
0.2
0.4
0.6
0.8
1.0
1.2
1.4
1.6
1.8
2.0
Frequency (THz)
Al_2O_3 with N_2
pristine
23.16 MGy

c

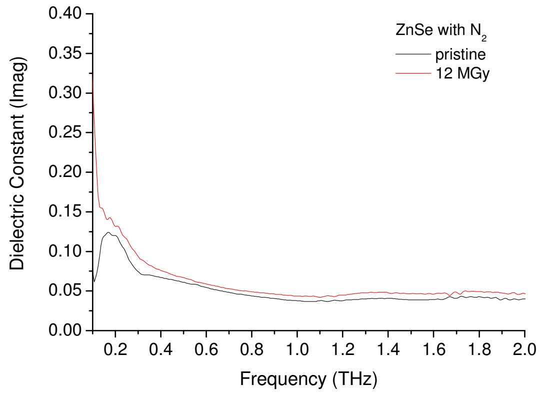

0.40
0.35
0.30
0.25
0.20
0.15
0.10
0.05
0.00
Dielectric Constant (Imag)
0.2
0.4
0.6
0.8
1.0
1.2
1.4
1.6
1.8
2.0
Frequency (THz)
ZnSe with N_2
pristine
12 MGy

d

Supplementary Figure 15. Irradiation induced changes of the dielectric constant imaginary part for: a – CaF_2; b – BaF_2; c – sapphire; d – ZnSe.

THz reflected signals obtained in the B-scan (along the selected *x* axis) and C-scan (the *xy* plane) modes are given in Supplementary Figs. 16 and 17.

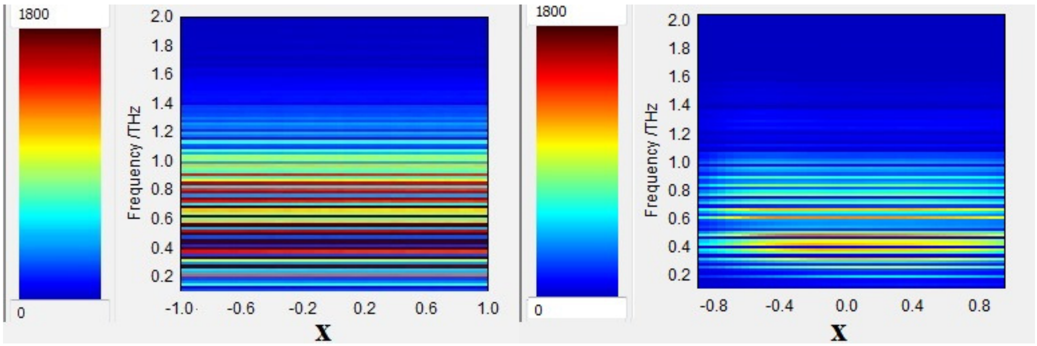

a b

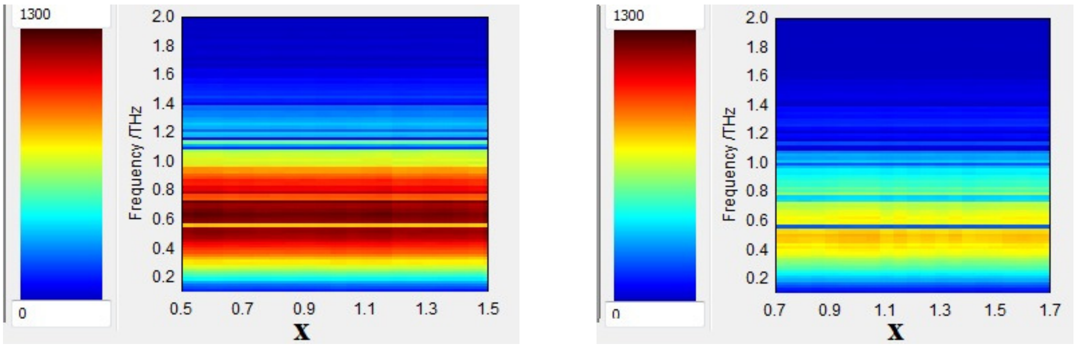

c d

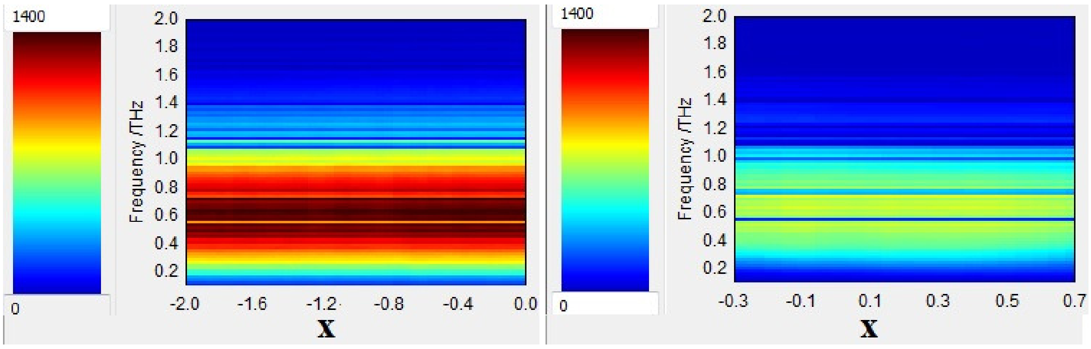

e f

Supplementary Figure 16. THz reflectance 2D image along the *x* axis as function of THz frequency (B-scan) using the frequency domain analysis mode for: a – BaF_2 pristine sample (detail); b – BaF_2 sample exposed up to the dose of 54.6 MGy (detail); c – Al_2O_3 pristine sample; d – Al_2O_3 sample exposed up to the dose of 5.79 MGy; e – ZnSe pristine sample; f – ZnSe sample exposed up to the dose of 6 MGy.

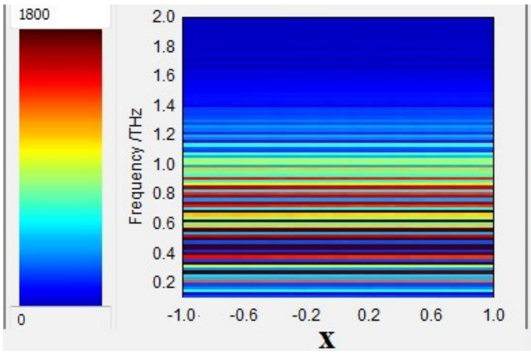

a

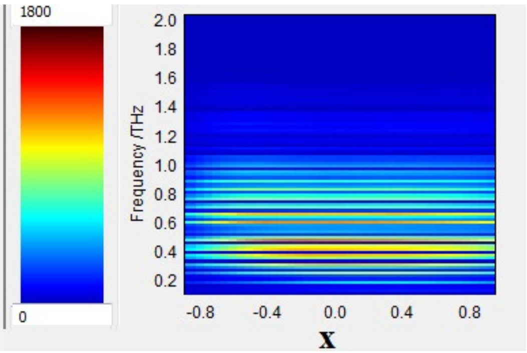

b

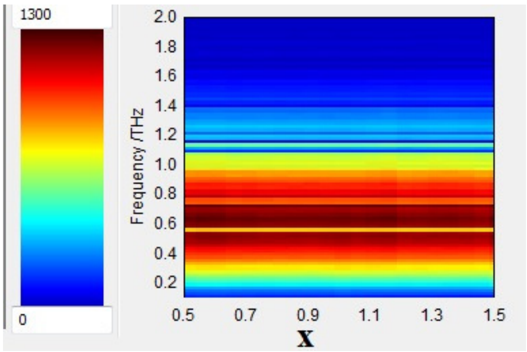

c

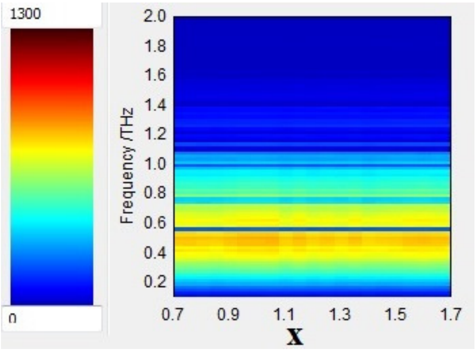

d

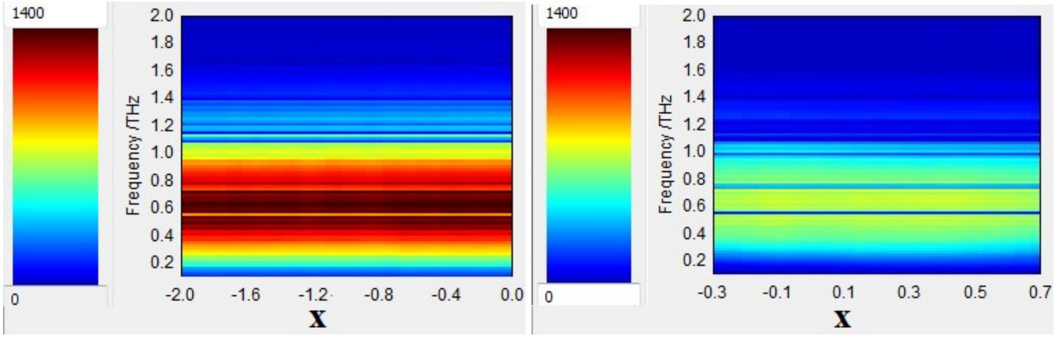

e f

Supplementary Figure 17. THz cross section in the *xy* plane (C-scan) using the frequency domain analysis mode for: a – BaF_2 pristine sample; b – BaF_2 sample exposed up to the dose of 54.6 MGy (zoom image); c – Al_2O_3 pristine sample; d – Al_2O_3 sample exposed up to the dose of 5.79 MGy; e – Zn Se pristine sample; f – ZnSe sample exposed up to the dose of 6 MGy.

Supplementary Fig. 18 offers information on a structural defect by THz imaging in the frequency domain analysis representation.

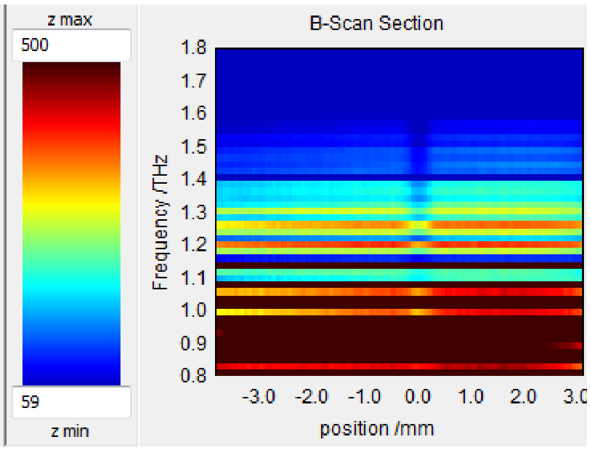

Supplementary Figure 18. THz reflected signal localizing a defect in the BaF_2 sample by using the frequency domain analysis.

The representation of the irradiation effects on the four windows as derived from the reflected THz signal represented in the time domain analysis mode are provided in Supplementary Fig. 19.

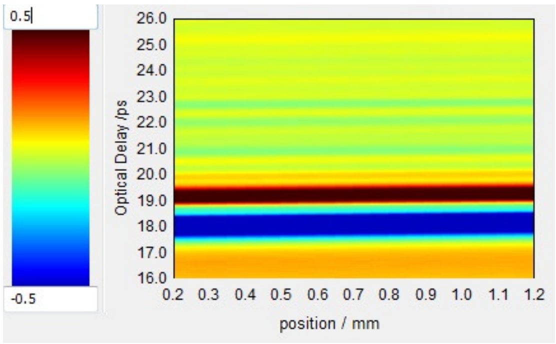

a

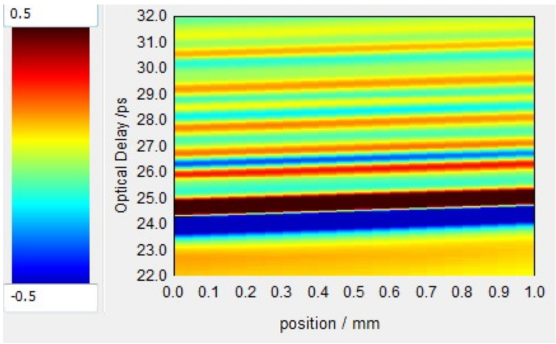

b

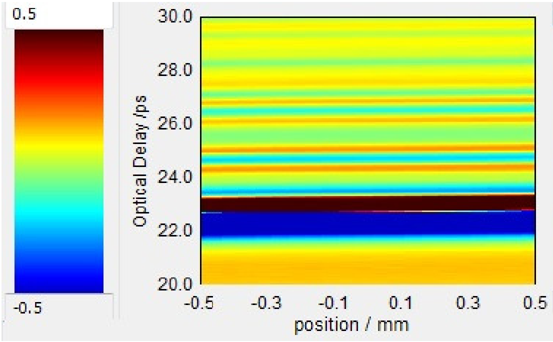

c

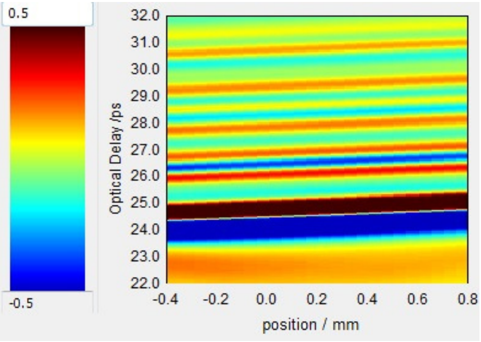

d

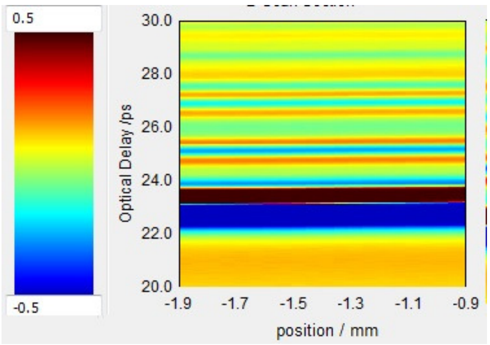

e

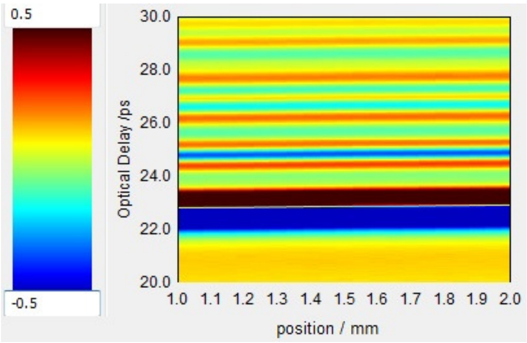

f

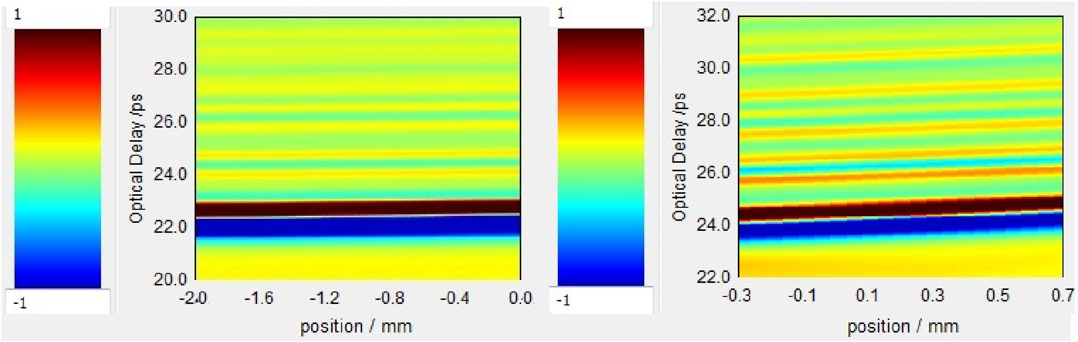

g h

Supplementary Figure 19. 2D representation of the THz reflected signal according to the time domain analysis for: a – CaF_2 pristine sample; b – CaF_2 sample irradiated at the dose of 8.76 MGy; c – BaF_2 pristine sample; d – BaF_2 sample irradiated at the dose of 54.6 MGy; e – Al_2O_3 pristine sample; f – Al_2O_3 sample irradiated at the dose of 5.79 MGy; g – Zn Se pristine sample; h – ZnSe sample irradiated at the dose of 6 MGy.

Investigated samples

Considering the incidence of some mid-IR materials used in spectroscopy applications we studied: CaF_2, BaF_2, ZnSe and sapphire (Supplementary Table 1) as they were exposed to alpha particle irradiation. For reader's convenience we included some data sheets characteristics typical for these materials. The irradiations were run in several subsequent steps, and, before and after each irradiation, their characteristics were measured in the optical and THz spectral range.

We included in the Table S1 general data concerning optical, mechanical and thermal specifications, as well as information about the density and molar mass of the investigated specimen, as they are of interest in evaluating each material's behaviour during the interaction with alpha particles, considering that: (i) the irradiation is performed in vacuum without assisted

cooling; (ii) the absorbed dose, the penetration depth and the degradation of the sample surface quality are depended on the characteristics of exposed material.

Optical material	Characteristics	Transparent spectral range (µm)	Sample diameter (mm)	Sample thickness (mm)	References
CaF_2	- Class/structure: cubic, (111) cleavage<br>- Density (g cm^{-3}): 3.18<br>- Molar mass (g mol^{-1}): 78.07<br>- Lattice constant (Å): 5.46<br>- Melting point (°C): 1418<br>- Thermal expansion coefficient (10^{-6}/K): 16.2-19.4<br>- Thermal conductivity (W m^{-1} K^{-1}): 9.17<br>- Specific heat capacity (J kg^{-1} K^{-1}): 888<br>- Hardness (Mohs): 4	0.2 – 13	10	1	[Supplementary1]
BaF_2	- Class/structure: cubic, (111) cleavage<br>- Density (g cm^{-3}): 4.83<br>- Molar mass (g mol^{-1}): 175.32<br>- Lattice constant (Å): 6.196<br>- Melting point (°C): 1354<br>- Thermal expansion coefficient (10^{-6}/K): 16.5-19.2<br>- Thermal conductivity (W m^{-1} K^{-1}): 7.1<br>- Specific heat capacity (J kg^{-1} K^{-1}): 456<br>- Hardness (Mohs): 3	0.2 – 16	11	1	[Supplementary 2]
Sapphire (Al_2O_3)	- Structure: hexagonal<br>- Density (g cm^{-3}): 3.98<br>- Molar mass (g mol^{-1}): 101.94<br>- Lattice constant (Å): a=4.785, c=12.991<br>- Melting point (°C): 2030<br>- Thermal expansion coefficient (10^{-6}/K): 3.24 - 5.66 (vertical c-axis)<br>- Thermal conductivity (W m^{-1} K^{-1}): 23.1 (vertical c-axis); 25.2 (parallel c-axis)	0.2 – 6.8	15	2	[Supplementary 3, Supplementary 4]

	- Specific heat capacity (J kg^{-1} K^{-1}): 761<br>- Hardness (Mohs): 9				
ZnSe	- Structure: cubic<br>- Density (g cm^{-3}): 5.27<br>- Molar mass (g mol^{-1}): 144.28<br>- Lattice constant (Å): 5.67<br>- Melting point (°C): 1520<br>- Thermal expansion coefficient (10^{-6}/K): 6.4 -7.6<br>- Thermal conductivity (W m^{-1} K^{-1}): 16.9<br>- Specific heat capacity (J kg^{-1} K^{-1}): 355<br>- Hardness (Mohs): 4	0.5 - 22	25	3	[Supplementary 5]

Supplementary Table 1. Characteristics of the investigated mid-IR optical materials.

Alpha particle irradiation and simulations

Four series of irradiation with 3 MeV alpha particles were performed at the U-120 Cyclotron accelerator, in operation at the Horia Hulubei" National Institute of Physics and Nuclear Engineering, having individual doses corresponding to a charge of: 100 μC, 200 μC, 300 μC, 300 μC. The equivalent accumulated total charges for each sample were in this case: 100 μC, 300 μC, 600 μC and 900 μC, respectively. Special mounts were designed to hold the windows during the irradiation as these were mounted in a XYZ micro positioning stage (travel range = 10 mm, travel resolution = 0.001 mm), which made possible the precise repositioning of the sample irradiated area, for subsequent irradiations. The alpha particle beam cross section (*A*), as measured by exposing a sensitive film to the beam, was appreciatively 6 mm^2. During the irradiations, the beam current was kept at 100 nA, while the focusing system current was 20 nA, in order to assure reproductively the beam diameter at the sample surface.

In Supplementary Table 2 are given the irradiation conditions for each type of mid-IR optical material and the corresponding irradiation doses. The volume (V) of optical material affected by the alpha particles is:

$$V = A * d\,, \qquad \text{(Supplementary1)}$$

where A is alpha particle beam cross section, and d denotes the penetration depth.

The mass (m) of the mid-IR optical particle affected by the interaction is:

$$m = V * \rho\,, \qquad \text{(Supplementary 2)}$$

where ρ designates the optical material density.

The equivalent dose (H) received by the sample upon an irradiation is computed as follows:

$$H = Q * E / m\,, \qquad \text{(Supplementary 3)}$$

E being the beam energy expressed in MeV.

Sample	Irradiation step	Density ρ (g/cm^3) Penetration depth d (µm) Mass m (g)	Beam charge Q (µC)	Fluence (α/cm^2)	Dose H (Gy)
CaF_2	1	3.18<br>9.08<br>$2.0\ 10^{-4}$	100	$0.9\ 10^{15}$	$1.46\ 10^{6}$
	2		200	$1.8\ 10^{15}$	$3.0\ 10^{6}$
	3		300	$2.7\ 10^{15}$	$4.38\ 10^{6}$
	4		300	$2.7\ 10^{15}$	$4.38\ 10^{6}$
BaF_2	1	4.83<br>11.2<br>$3.28\ 10^{-4}$	100	$0.9\ 10^{15}$	$9.1\ 10^{6}$
	2		200	$1.8\ 10^{15}$	$18.2\ 10^{6}$
	3		300	$2.7\ 10^{15}$	$2.7\ 10^{7}$
	4		300	$2.7\ 10^{15}$	$2.7\ 10^{7}$
Sapphire	1	3.98<br>6.5<br>$1.55\ 10^{-4}$	100	$0.9\ 10^{15}$	$1.93\ 10^{6}$
	2		200	$1.8\ 10^{15}$	$4.0\ 10^{6}$
	3		300	$2.7\ 10^{15}$	$5.79\ 10^{6}$
	4		300	$2.7\ 10^{15}$	$5.79\ 10^{6}$
ZnSe	1	5.27<br>9.5<br>$3.0\ 10^{-4}$	100	$0.9\ 10^{15}$	$1.0\ 10^{6}$
	2		200	$1.8\ 10^{15}$	$2.0\ 10^{6}$
	3		300	$2.7\ 10^{15}$	$3.00\ 10^{6}$
	4		300	$2.7\ 10^{15}$	$3.00\ 10^{6}$

Supplementary Table 2. Details of the irradiation conditions for the four mid-IR investigated optical materials.

For on-line radioluminescence measurements, a UV-visible 400 μm core diameter multimode optical fiber was installed in the experimental vacuum chamber (Supplementary Fig. 20) and was coupled by a vacuum feedthrough to a high sensitive QE65000 Ocean Optics optical fiber spectrometer. Radioluminescence data were collected using the spectrometer native Spectral Suite software. The box car and the average values were 1, while the integration time was 10 s (ZnSe), and 20 s (CaF_2, BaF_2, Al_2O_3). The spectra were acquired over the spectral range from 200 nm to 1 μm.

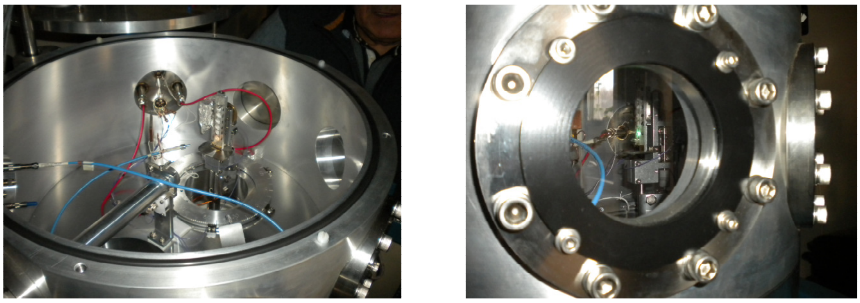

Supplementary Figure 20. The optical fiber-based setup for on-line, under vacuum, radioluminescence monitoring during alpha particle irradiation.

The SRIM application was used to estimate the alpha particle beam penetration in each mid-IR optical material [Supplementary 6].

For each sample an elementary RBS (Rutherford backscattering spectrometry) analysis was performed, using SIMNRA application [Supplementary 7], to establish the composition of the respective sample. For this investigation RC43 NEC End Station instrument was used [Supplementary 8].

Optical investigations

The optical investigations were performed prior to irradiation and after each irradiation step and consist of:

- visual and microscopic inspection of the samples for irradiation induced color change and radiation induced window's surface roughness modification;
- spectral measurements of the optical transmittance and optical diffused reflectance, over a wide spectral range, appropriate to each investigated optical material.

The spectral measurements were performed with the Gooch & Housego double monochromator OL Series 750 Automated Spectroradiometric Measurement System. The setups used for the measurements run over different spectral intervals are detailed in Supplementary Table 3.

Spectral range (μm)	Light source	Grating	Slit/ aperture	Optics module	Detector
**0.2 – 1.8**	Tungsten	Turret 1 (0.25 μm blaze, 1200 g/mm; 0.5 μm blaze, 600 g/mm; 1.6 μm blaze, 600 g/mm)	2.5 mm – 5 mm/ 3 mm	PTFE sphere	**Si & Ge**
**1.8 – 2.1**					**PbS**
**2.1 – 3.2**		Turret 2 (4.0 μm blaze, 150 g/mm; 10 μm blaze, 75 g/mm; 22.5 μm blaze, 40 g/mm)		Gold sphere	
**3 – 5**	IR glower				**InSb**
**5 – 18**					**HgCdTe**

Supplementary Table 3. The detection setups used for transmittance and reflectance spectral measurements.

THz spectral investigations THz imaging

The TeraView TPS Spectra 3000 spectrometer was used with the Transmittance and the Reflectance Imaging Module. Supplementary Figure 3 provide information on the THz reflectance based imaging geometry, indicating the plans or axis used to represent the THz detected signal prior and post irradiation.

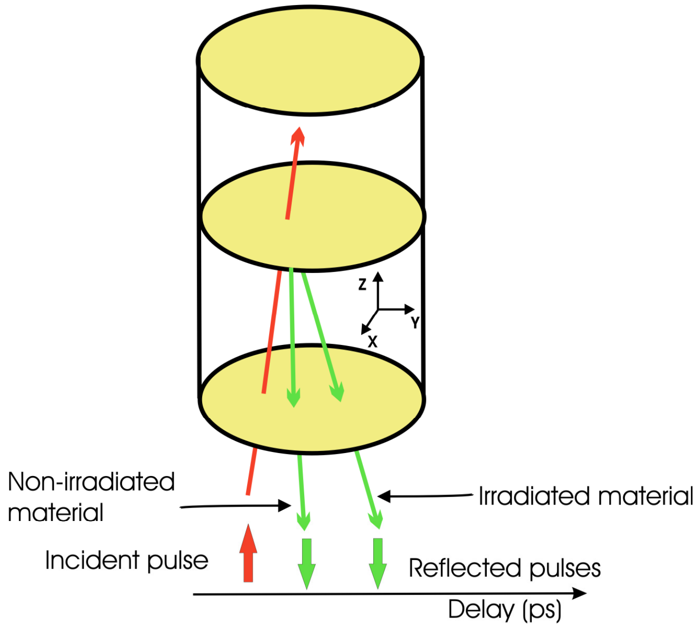

Supplementary Figure 21. The detection geometry used in THz reflectance imaging. The change of material properties upon the irradiation produces a delay in the detected pulse as referred to the interrogation pulse.

References for supplementary materials

Supplementary 1. http://crystal-gmbh.com/shared/downloads/datenblaetter/optics_de/Kalziumfluorid_Calcium_Fluoride_ CaF2.pdf.

Supplementary 2. http://crystal-gmbh.com/shared/downloads/datenblaetter/optics_de/Bariumfluorid_Barium_Fluoride_BaF2.pdf.

Supplementary 3. http://crystal-gmbh.com/shared/downloads/datenblaetter/optics_de/Saphir_Sapphire _Al2O3.pdf.

Supplementary 4. Dobrovinskaya, E. R., Lytvynov, L. A. & Pishchik, V. *Sapphire: material, manufacturing, applications*. Ch. 2, 55-175 (Springer Science & Business Media, 2009).

Supplementary 5. http://crystal-gmbh.com/shared/downloads/datenblaetter/optics_de/Zinkselenid_Zinc_ Selenide_ZnSe.pdf.

Supplementary 6. www.SRIM.org.

Supplementary 7. Mayer, M., SIMNRA, a simulation program for the analysis of NRA, RBS and ERDA. In *The fifteenth international conference on the application of accelerators in research and industry*, AIP Publishing, **475**, 541-544 (1999).

Supplementary 8. http://www.pelletron.com/necold5.htm#RC43.
